# Evaluation of Different Tandem MS Acquisition Modes to Support Metabolite Annotation in Human Plasma Using Ultra High-Performance Liquid Chromatography High-Resolution Mass Spectrometry for Untargeted Metabolomics

**DOI:** 10.3390/metabo10110464

**Published:** 2020-11-15

**Authors:** Julian Pezzatti, Víctor González-Ruiz, Julien Boccard, Davy Guillarme, Serge Rudaz

**Affiliations:** 1 School of Pharmaceutical Sciences, University of Geneva, Rue Michel-Servet 1, 1211 Geneva 4, Switzerland; julian.pezzatti@unige.ch (J.P.); victor.gonzalez@unige.ch (V.G.-R.); julien.boccard@unige.ch (J.B.); davy.guillarme@unige.ch (D.G.); 2 Institute of Pharmaceutical Sciences of Western Switzerland, University of Geneva, 1211 Geneva 4, Switzerland; 3 Swiss Centre for Applied Human Toxicology (SCATH), 4055 Basel, Switzerland

**Keywords:** metabolomics, ultra-high performance liquid chromatography, high-resolution mass spectrometry, ion mobility mass spectrometry, tandem mass spectrometry, metabolite annotation, human plasma

## Abstract

Ultra-high performance liquid chromatography coupled to high-resolution mass spectrometry (UHPLC-HRMS) is a powerful and essential technique for metabolite annotation in untargeted metabolomic applications. The aim of this study was to evaluate the performance of diverse tandem MS (MS/MS) acquisition modes, i.e., all ion fragmentation (AIF) and data-dependent analysis (DDA), with and without ion mobility spectrometry (IM), to annotate metabolites in human plasma. The influence of the LC separation was also evaluated by comparing the performance of MS/MS acquisition in combination with three complementary chromatographic separation modes: reversed-phase chromatography (RPLC) and hydrophilic interaction chromatography (HILIC) with either an amide (aHILIC) or a zwitterionic (zHILIC) stationary phase. RPLC conditions were first chosen to investigate all the tandem MS modes, and we found out that DDA did not provide a significant additional amount of chemical coverage and that cleaner MS/MS spectra can be obtained by performing AIF acquisitions in combination with IM. Finally, we were able to annotate 338 unique metabolites and demonstrated that zHILIC was a powerful complementary approach to both the RPLC and aHILIC chromatographic modes. Moreover, a better analytical throughput was reached for an almost negligible loss of metabolite coverage when IM-AIF and AIF using ramped instead of fixed collision energies were used.

## 1. Introduction

Untargeted metabolomics constitutes a potent approach to assess phenotypic modifications at the molecular level caused by disease, drug efficacy or toxicity, environmental factors, etc. Thus, it is a science of prime importance to tackle and understand the complex area of human personalized medicine [1,2,3,4]. In untargeted metabolomics, it is necessary to monitor the broadest possible range of metabolites reproducibly and to retrieve as much chemical information as possible. The large molecular coverage of untargeted approaches enables the discovery of diverse metabolites involved in different biological pathways and allows researchers to characterize pathologies such as chronic kidney disease, diabetes, or cancer by providing a diagnostic chemical signature of the related metabolic phenotypes [2,5,6,7,8].

It is now well-recognized within the scientific community that obtaining broad metabolic coverage can only be achieved through a combination of different analytical platforms [9,10,11,12,13,14,15]. For this purpose, we recently proposed a strategy comprising three complementary LC methods, including reversed-phase chromatography (RPLC) and hydrophilic interaction chromatography with amide (aHILIC) and polymeric zwitterionic (zHILIC) stationary phases, all of which are coupled with high-resolution mass spectrometry (HRMS) [16]. In fact, thanks to its hydrophilic partitioning, hydrogen bonding, ion exchange, and dipole–dipole interactions, HILIC is a chromatographic mode that is perfectly adapted for the analysis of very polar compounds, while RPLC is well suited for retaining moderately polar to apolar metabolites [17,18].

Metabolite annotation is still one of the main challenges in untargeted metabolomics. Nevertheless, thanks to the continuous developments of quadrupole time of flight (Q-ToF) and Orbitrap mass analyzers, investigators now have access to a relatively enhanced dynamic range and acquisition rate, excellent mass accuracy, improved sensitivity, as well as strategies for performing multi-event acquisitions, enabling concomitant metabolite detection, relative quantification, and structural characterization through MS/MS experiments [19]. Although the development of in-house databases based on measuring the properties of authentic standards allows for the identification of a large number of metabolites in many studies, the biological information gathered by this approach is limited by the availability and cost of the chemical standards [20,21,22]. To increase metabolic knowledge, publicly available MS/MS mass spectral and collision cross-section (CCS) databases can be used [23,24,25,26]. In fact, the available MS/MS spectral libraries, such as HMDB (www.hmdb.ca/), Metlin (metlin.scripps.edu/), NIST (www.nist.gov/srd/nist-standard-reference-database-1a-v17), MoNA (mona.fiehnlab.ucdavis.edu/), GNPS (gnps.ucsd.edu/), LipidMaps (www.lipidmaps.org/), and CMM (ceumass.eps.uspceu.es), are by far the most widely used resources because they allow for fast and usually inexpensive metabolite annotation of the generated data [22,27,28,29,30,31].

Tandem MS can be performed either by data-dependent analysis (DDA, also called data-directed analysis) and all ion fragmentation (AIF), which is a data-independent analysis mode (DIA, also called MS^E^ in Waters instruments) [32,33]. DDA can be performed by collecting a survey scan, and then, the top *n*-most intense precursor ions are selected within their respective narrow *m/z* windows before fragmentation takes place. This acquisition mode provides clean MS/MS spectra, and the product ions are easily related to their precursors. However, due to the relatively short LC analysis time and the limited instrument duty cycle, the MS/MS spectra will not be collected for all detected metabolites [19,34,35]. To overcome this limitation, the use of intelligent exclusion lists, in which already fragmented metabolites from an initial analytical run are excluded from the fragmentation candidates in a second DDA run, enables the fragmentation of less intense metabolites [35]. Alternatively, AIF is performed by fragmenting all the available ions (without selecting precursors) when no *m/z* window is selected over the whole precursor *m/z* range [19,35]. This acquisition mode enables the operators to fragment all the precursor ions, but the major limitation is the difficulty in linking each fragment to its precursor, leading to identification inaccuracies. These difficulties can occur more frequently when trying to identify coeluting isobaric compounds (i.e., those with the same retention time, peak shape, peak apex, and *m/z*). To overcome this limitation, the use of deconvolution algorithms to reconstruct the pure MS/MS spectrum can be used [32,35]. Moreover, the MS instruments’ peak capacity and dynamic range have been increased, and the performance of AIF has been improved with ion mobility-high resolution mass spectrometry (IM-HRMS) instrumentation [36,37].

With the advent of the latest technical breakthroughs in MS devices, ion mobility spectrometry (IM) coupled to HRMS instruments are becoming increasingly popular in many fields, such as food sciences, biopharmaceutical analysis, forensics as well as metabolomics, lipidomics, and others [36,38,39,40,41,42,43]. Notably, IM represents a complementary and powerful analytical technique to improve the identification and characterization of analytes of interest [44]. IM is a post-ionization technique, which entails separating ions according to their size, shape, and charge in the gas phase under the influence of an electric field. Different types of commercially available IM technologies allow investigators to obtain CCS values, which offer additional structural information about the detected metabolites, i.e., drift tube ion mobility spectrometry (DTIMS), travelling wave ion mobility spectrometry (TWIMS), trapped ion mobility spectrometry (TIMS), and differential mobility analyzers (DMA) [39]. DTIMS (i.e., the stepped field CCS method) is classified as a primary IM method in which the measured drift times can be directly derived into CCS values. DTIMS (i.e., a single-field CCS method), TWIMS, and TIMS are classified as secondary IM methods and require an instrumental calibration using reference compounds to determine the CCS values. Unlike the drift times, the CCS values are not instrument-dependent, and therefore, they should be comparable between different instruments and/or laboratories operating under the same experimental conditions, thus allowing the use of CCS external databases (referring to apparent CCS values) [26,45].

The aim of the present study was to compare the performance of diverse tandem MS acquisition modes, with and without IM, to annotate the metabolites in human plasma. The different evaluated tandem MS acquisition modes, namely, DDA or AIF with or without IM (known as HDDA, DDA, HDMS^E^, or MS^E^, respectively) [33] were investigated systematically. Furthermore, each mode was evaluated with different collision energy conditions, i.e., either with a ramp (10–60 eV and 30–60 eV) or fixed values (14, 28, and 56 eV). The influence of the LC separation on the overall peak capacity of each method was also evaluated by comparing the performance of the acquisition modes in combination with three previously developed complementary chromatographic approaches: RPLC and HILIC with either an amide (aHILIC) or a zwitterionic (zHILIC) stationary phase. This methodological comparison was conducted on a Standard Reference Material (NIST SRM 1950 human plasma) with the help of a mass spectral library containing both MS/MS and traveling-wave CCS information acquired on N_2_ (^TW^CCS_N2_) [36].

## 2. Results and Discussion

In untargeted metabolomics studies, UHPLC-(IM)-HRMS techniques yield a wealth of information presented as multi-dimensional arrays of measured intensities over *m/z*, Rt, and CCS values/drift times (when available). Metabolite annotation can be performed only after a series of data pre-processing steps involving peak picking and adduct deconvolution to obtain lower dimensionality tables containing intensities for each feature on each sample. This study reports the first systematic investigation on different tandem MS acquisition modes by UHPLC-IM-HRMS with a focus on untargeted metabolomics applications supported by three complementary chromatographic separation modes, i.e., RPLC, aHILIC, and zHILIC. To our knowledge, very few articles have evaluated different tandem MS approaches to identify drug metabolites and endogenous metabolites using a UHPLC-HRMS platform [34,46]. 

Biofluids such as plasma, serum, and urine are the most frequently collected human samples in metabolomics experiments because they provide a general overview of metabolism operating in various different compartments of the human body (organs, tissues, etc.) and are relatively easy to collect [47]. National Institute of Standards and Technology (NIST) human plasma (SRM 1950) was selected for this study because it is widely used as a reference material. To evaluate the obtained metabolite annotation, the Waters Metabolic Profiling CCS Library was chosen to match the MS/MS and ^TW^CCS_N2_ information acquired on a similar IM-HRMS instrumentation platform. In this study, the library was used as a benchmark to support our major aim, which was to challenge the different tandem MS acquisition modes. Last, the systematic experimental design employed here enabled a sequential evaluation of the investigated tandem MS acquisition modes, combining various factors (Table 1). First, in one chromatographic mode (i.e., RPLC), the usefulness of IM in metabolite annotations and the molecular knowledge afforded by DDA and AIF as well as by the collision energy strategies (ramped and fixed) was evaluated. Second, the selectivity improvement afforded by each one of the LC modes was assessed to select the most promising tandem MS methodologies.

### 2.1. Investigation of All Tandem MS Modes under RPLC Conditions

The RPLC conditions were first chosen to investigate all the tandem MS modes of the present study because this is the most widespread chromatographic mode in LC-based untargeted metabolomics due to its high robustness, versatility, and peak capacity. To improve the repeatability in the evaluation of all the tandem MS modes, conditioning and system suitability test (SST) samples were included at the beginning of each analytical sequence. These sequences comprised samples acquired with the different tandem MS modes described in Table 1. For each DDA analytical sequence, a second sequence was acquired, setting up an exclusion list containing ions already fragmented in the first round (Supplementary Tables S1 and S2).

From the raw datasets, approximately 15,800 features were obtained after peak picking and peak grouping. This number dropped to 1800 when activating the IM cell. This observation can be easily explained in that electric fields at higher voltages are applied to the lenses of the ion mobility cell when performing IM analyses (i.e., HDDA and HDMS^E^). Therefore, the loss of many ion signals (in this case) are encountered when performing AIF and DDA with IM analyses compared to classical AIF and DDA (without IM). To evaluate if the missing features shared any valuable property (common mass or ^TW^CCS_N2_ ranges or low-abundance species), we looked at the metabolite annotations retrieved for each investigated analytical condition.

By accounting for all the annotated metabolites using the accurate mass-^TW^CCS_N2_-MS/MS, accurate mass-^TW^CCS_N2_, accurate mass-MS/MS, and accurate mass only, 400–700 and 600–800 features could be annotated for the analyses performed here with and without IM, respectively (data not shown). No significant differences were found when comparing the metabolite annotations for these two modalities. When comparing two pairs of analytical runs, i.e., AIF ramp 10–60 and DDA ramp 10–60, with and without IM, we confirmed that features, corresponding to low-intensity ion signals, were lost when the IM cell was activated. However, this participates up to the IM capacity to provide cleaner mass spectra. 

Numerous putative metabolite annotations can be made in the list of filtered features based on the applied tolerance criteria, i.e., 5 ppm for precursor and fragment masses and 2.5% in the case of ^TW^CCS_N2_ [26,36]. For each annotated metabolite retrieved under one tandem MS acquisition mode, a search in the list of filtered features was performed to determine how many annotation hits were found for the same metabolite. In most cases, only one or two of them were obtained from the corresponding filtered features list (Supplementary Figure S1). This result allows for a fairly high level of confidence in the performed metabolite annotations, but it is related to the fact that only one database was used for annotation here. In the case of multiple matching, one could expect that the number of candidates will increase dramatically. Regarding metabolite annotations, only univocal matches (one single feature assigned to one single putative identity, without duplicate features or identities) were considered in the following sections. 

**Figure 1:**
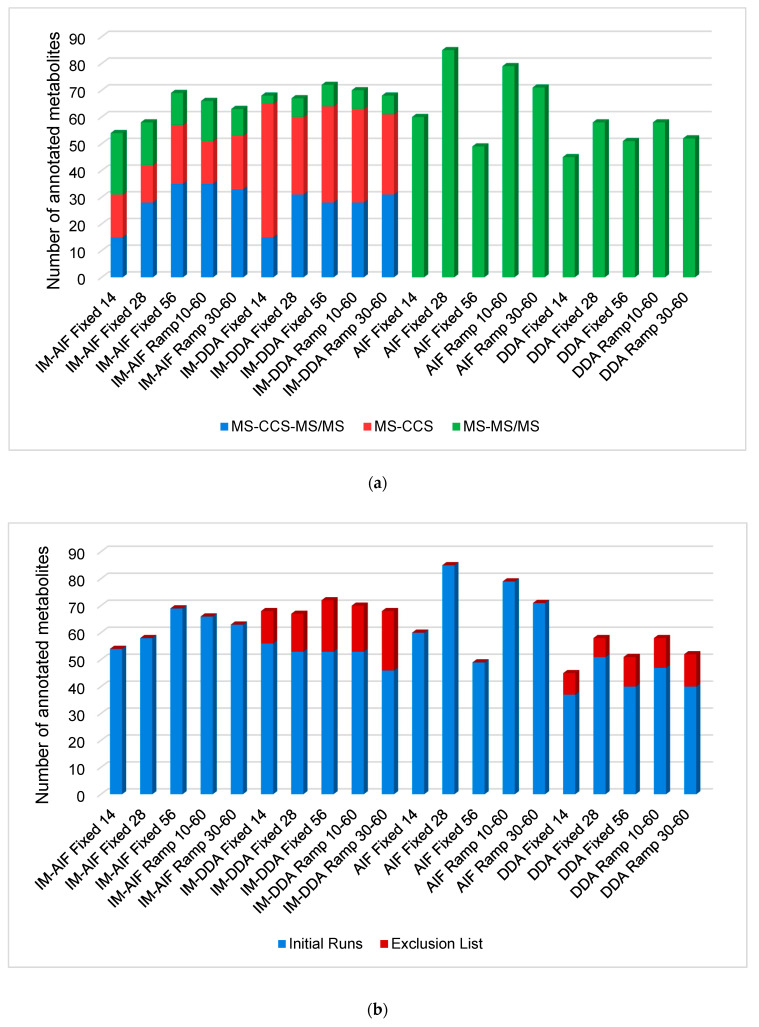
Overall unambiguous metabolite annotations (**a**) made by accurate mass-^TW^CCS_N2_-MS/MS (blue), accurate mass-^TW^CCS_N2_ (red) and accurate mass-MS/MS (green); (**b**) made during the initial runs (blue) and after the exclusion list runs (red).

From all the tandem MS modes investigated here, it was possible to annotate 186 unique metabolites either by accurate mass-^TW^CCS_N2_-MS/MS, accurate mass-^TW^CCS_N2_, or accurate mass-MS/MS depending on the acquisition mode specificities (Figure 1a). When the IM is on, it is possible to manage an additional molecular descriptor, namely, the ^TW^CCS_N2_, allowing the operator to annotate a significant number of metabolites by accurate mass-^TW^CCS_N2_. These metabolites were not annotated by accurate mass-^TW^CCS_N2_-MS/MS because of their low intensity, i.e., they were not selected for MS/MS in DDA acquisition modes (Figure 1a).

**Figure 2:**
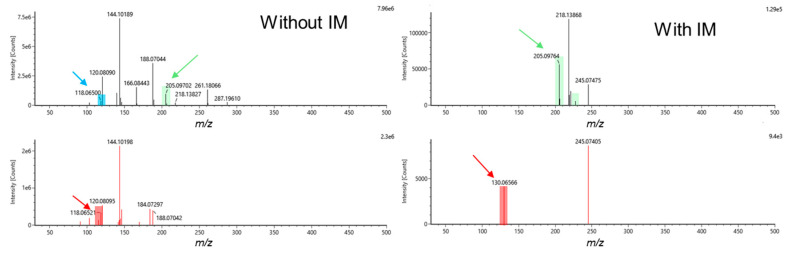
Example of MS and MS/MS spectra for tryptophan (M+H)^+^ in hydrophilic interaction chromatography with amide (aHILIC) electrospray ionization (ESI)+ for all ion fragmentation (AIF) ramp 10–60 eV with and without IM. The precursor ion *m/z* 205.0976 (green), in-source fragment *m/z* 118.0650 (blue), and fragment ions (red) *m/z* 118.0652 (AIF) and *m/z* 130.06566 (IM-AIF) were retrieved by the UNIFI software. In-source fragment at *m/z* 118.0650 is filtered out when the IM cell is activated.

In DDA with IM experiments, fewer metabolites were annotated by accurate mass-^TW^CCS_N2_-MS/MS and/or accurate mass-MS/MS compared to AIF with IM experiments. This results from the selection of the top 3 ions for MS/MS, and therefore, far fewer ions were fragmented, even when combining metabolite annotations from the initial runs and the second runs with the exclusion list for DDA modalities (Figure 1b). However, considering that the ^TW^CCS_N2_ values are correlated to the *m/z*, accurate mass-^TW^CCS_N2_ annotations have to be taken as a lower confidence level of annotation than accurate mass-MS/MS annotations, which involves two orthogonal properties [48,49]. Moreover, as already discussed, a significant loss of low intensity features was encountered when performing analyses with IM, making it difficult to detect and/or fragment low-intensity metabolites. When looking at the two runs performed for DDA analyses (initial run and with exclusion lists), it was observed that the second run brings a significant number of additional metabolite annotations, corresponding to approximately 20–25% of the total annotated metabolites (Figure 1b). By taking into account the amount of resources needed for this additional gain of information, one could question the real added value. It appears that for DDA with IM analyses, it is possible to annotate metabolites similarly to AIF with IM acquisition modes, but with a larger proportion of metabolites annotated by accurate mass-^TW^CCS_N2_. However, for DDA without IM analyses, far fewer metabolites can be annotated compared to the AIF acquisition modes (Figure 1b). Although DDA acquisition modes normally provide cleaner precursor and fragment mass spectra compared to AIF, the added IM values already provide high-quality spectra, thus preventing false positive annotations, as represented by the example of tryptophan given in Figure 2. In AIF with IM acquisition modes, IM allows for the separation of LC coeluting precursor ions in the drift time dimension before fragmentation takes place. Therefore, precursor ions coeluting at the same retention time can be separated in the IM dimension, and fragments that do not match the precursor ion’s drift time can be filtered out, as highlighted in Figure 2. However, in-source fragments of metabolites of interest will also be filtered out, as it is shown for the in-source fragment of tryptophan (*m/z* 188.0650), which is present in AIF mode but is absent when IM is used. Moreover, it is important to note that the IM’s ability to clean precursor and fragments mass spectra is balanced with the loss of lower intensity metabolites, as already demonstrated for analyses using capillary electrophoresis IM-HRMS [44]. Notably, for tryptophan (Figure 2), the intensity was 16 times lower when an AIF with IM analysis was performed compared to AIF alone (943,707 counts in AIF and 56,427 counts in IM-AIF).

**Figure 3:**
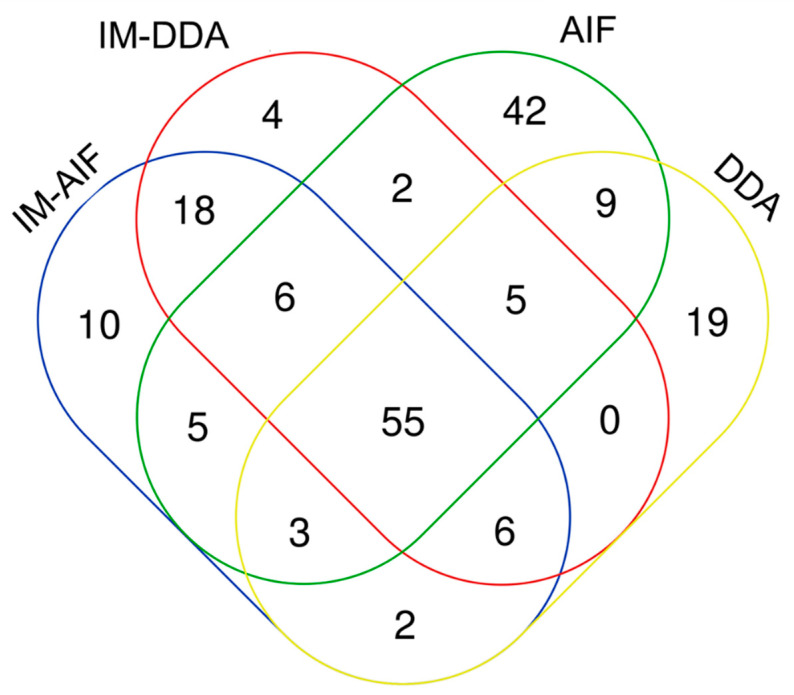
Overall metabolite annotation obtained using IM-AIF, IM-DDA, AIF, and data-dependent analysis (DDA) modalities.

When comparing overall metabolite annotations by AIF and DDA with and without the IM acquisition modes, 55 metabolites could be annotated with each of the four modalities (Figure 3). These metabolites belong to different chemical families and sub-families such as carnitines, organic acids, steroids, and lipids (lysophospholipids and phosphatidylcholines) (Supplementary Figure S2). A significant portion of features were annotated only through AIF acquisitions without IM (42 metabolites) and to a lesser extent through DDA (only 19 metabolites). The 42 annotated metabolites by AIF without IM acquisitions belong to the organic acids, steroids, amino acids, and lipids (phosphatidylcholines, phosphatidylserines, and lysophospholipids) (Supplementary Figure S3). These metabolites could likely be annotated only with the AIF acquisition modes because they were found at very low concentrations in the treated samples, and they could not be detected when the IM cell was activated. By contrast, DDA with IM (i.e., HDDA) acquisition did not provide any specific chemical information compared to the other evaluated modes (Figure 3).

On average, tandem MS acquisition modalities using ramped or fixed collision energies allowed annotating the same number of metabolites. AIF with and without IM analyses using ramps provided annotations of fewer metabolites than when fixed collision energies were used, i.e., from 5 to 10 metabolites. Indeed, 13 vs. 25% and 16 vs. 24% exclusive compounds were annotated in AIF with IM ramped vs. fixed and AIF only ramped vs. fixed, respectively (Supplementary Figure S4). Therefore, it is interesting to see that with two analytical runs using ramps, it is possible, on average, to annotate as many metabolites as you can with three analyses using fixed energies, thus reducing the analysis time and the number of biological samples needed.

From this study, it appears that DDA with exclusion lists (1) does not provide a significant additional amount of chemical coverage, (2) is more time-consuming with two analytical sequences for reaching comparable annotation rate, and (3) cleaner MS/MS spectra can be obtained by IM when performing AIF acquisitions (i.e., HDMS^E^); therefore, the other LC methods were evaluated with only two acquisition modes, i.e., AIF with and without IM. 

Since the peak capacity of each LC-MS analytical method depends on the ability of the LC separation to provide fewer coeluting peaks to the MS for analysis and fragmentation, the potential of different LC separation modes (aHILIC and zHILIC) to improve the overall performance of the analytical platform was first investigated.

### 2.2. Evaluating the Best Tandem MS Modes for HILIC Separations

Both HILIC chromatographic methods were chosen by accounting for their ability to analyze a wide chemical range of polar metabolites such as amino acids, nucleosides, and carnitines by aHILIC, and nucleotides, organic acids, and phosphorylated sugars by zHILIC [16,50]. As previously mentioned, and based on the results obtained in RPLC mode, only AIF with and without IM acquisition modes were investigated for HILIC metabolite annotation. In this case, we also investigated the difference between ramped and fixed collision-induced dissociation (CID) modalities. The latter is generally performed at three different energies (low, medium, and high) [22]. This strategy takes account of the lability of metabolites. In fact, excessively low or high collision energies could lead to an absence or excess of metabolite fragmentation. Considering that tryptophan was annotated in aHILIC, MS/MS fragmentation set at fixed 14 and 56 eV CID led to low and excess fragmentation, respectively. However, with a ramped MS/MS acquisition of 10–60 eV, an optimal fragmentation could be reached, but in this case, transmitted ions will spend much less time per collision energy range (low, medium, and high) compared to fixed collision energies, since during each fragmentation cycle, time is spent sweeping the chosen desired range. Therefore, we aimed to compare the annotation performance of three different fixed collision energies to the use of a ramp of energies and the throughput gain when using one or two analytical runs instead of three. 

Taking into account all the investigated AIF acquisition modes, 187 and 177 unique metabolites were annotated either by accurate mass-^TW^CCS_N2_-MS/MS, accurate mass-^TW^CCS_N2_, or accurate mass-MS/MS, depending on the acquisition mode specificities for aHILIC and zHILIC, respectively. This time, significantly more metabolites were annotated by ramp acquisition modes compared to fixed dissociation energies for both chromatographic methods (Supplementary Figures S5 and S6). Once again, more metabolites were annotated by AIF alone compared to AIF with IM because of the low abundance metabolites, which could not be detected or annotated. 

**Figure 4:**
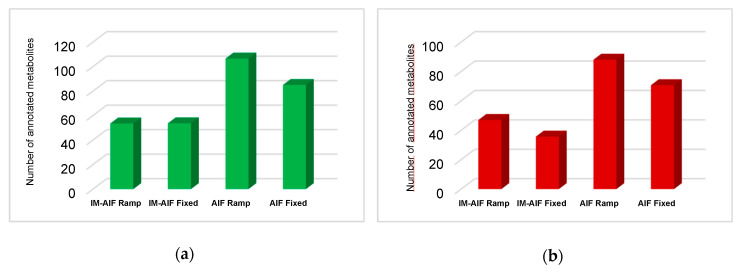
Average metabolite annotations by IM-AIF ramped and fixed CID and AIF ramped and fixed CID for (**a**) aHILIC ESI+ (green); and (**b**) zHILIC ESI- (red).

To obtain a deeper knowledge of how ramped collision energies behave against fixed ones, the average annotations made with AIF with/without IM and ramped and fixed modalities were compared (Figure 4). On average, AIF with/without IM using ramped energies allowed for the annotation of more metabolites than fixed collision energies. Moreover, ramped modalities cover a similar number and even some more specific metabolites (Supplementary Figures S7 and S8). Only a low number of exclusive metabolites were annotated by fixed collision energies under zHILIC conditions. Similar conclusions were also obtained for the RPLC conditions. Even though ions spend much less time per collision energy range (low, medium, and high) during ramped MS/MS acquisitions, a majority of the metabolites could be detected and annotated. 

To save time and volume of valuable biological samples, we recommend using one or two MS/MS ramp acquisitions when confirming metabolite identities during untargeted metabolomics experiments. The first investigated one, at 10–60 eV, was found to be appropriate for relatively labile compounds, while the second one, at 30–60 eV, was suitable for compounds that are more difficult to fragment. Since it is not possible, in untargeted metabolomics, to anticipate the chemical nature of discriminating metabolites, we advise the application of both ramped acquisition modes without IM (i.e., MS^E^ modality) during two successive runs at the beginning (right after the conditioning runs) or at the end of the analytical sequence. Only if the quantity of biological sample is sufficient can additional analyses with IM (i.e., HDMS^E^ modality) be considered.

### 2.3. Optimal Combination of LC and MS/MS Modes to Improve Human Plasma Metabolome Coverage

**Figure 5:**
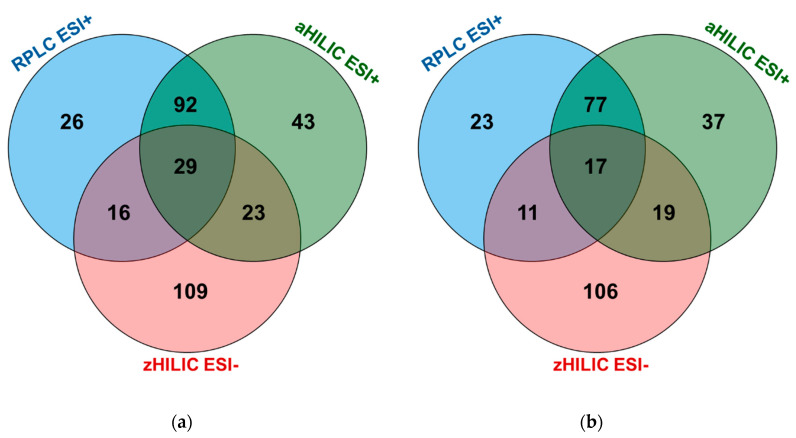
Overall metabolite annotations for RPLC ESI+, aHILIC ESI+, zHILIC ESI- with (**a**) all IM-AIF and AIF acquisition modes; and (**b**) IM-AIF and AIF ramped 10–60 and 30–60 eV acquisition modes.

To ensure consistent comparisons, only metabolites annotated in RPLC, aHILIC, and zHILIC by AIF with and without IM modalities will be discussed here. The DDA acquisition modes will not be investigated. Totals of 163, 187, and 177 unique metabolites were annotated by all investigated AIFs with and without IM modalities for RPLC, aHILIC, and zHILIC, respectively. The list of annotated metabolites for each LC technique and tandem MS modality, along with various information comprising the PubChem ID and name, Rt, *m/z*, ^TW^CCS_N2_ (when available), chemical class, MS/MS mode(s), overall score, fragmentation score, mass error (ppm), isotope similarity, and delta CCS are given in Supplementary Tables S3–S5. Since some metabolites have been annotated in several tandem MS acquisition modes at the same retention time using the same chromatographic separation mode, it is possible for researchers to use this information to annotate metabolites from future metabolomics studies, allowing the expansion of in-house databases. By addressing the fact that annotations of several metabolites could be obtained under different conditions, 338 univocally annotated compounds were ultimately obtained from human plasma NIST SRM 1950 (Figure 5a). This number is 35% higher than a recent study about an inter-laboratory ring trial for human serum and human, mouse, and rat plasma (including NIST SRM 1950) samples using RPLC- and flow injection analysis (FIA)-HRMS. Indeed, in this research, 250 polar and apolar metabolites were routinely detected by 14 laboratories [51].

It is interesting to evaluate the complementarity offered by the different LC methods. First, zHILIC was confirmed to be a powerful complementary separation approach to both the RPLC and aHILIC chromatographic modes. The number of specific metabolites detected by zHILIC is indeed the largest (Figure 5a). Then, we observed that only a few metabolites were commonly annotated between either RPLC and zHILIC (16 common metabolites) or aHILIC and zHILIC (23 common metabolites) (Figure 5a). By contrast, the complementarity between RPLC and aHILIC was more limited (92 common metabolites). This peculiarity of zHILIC has already been demonstrated and evaluated in a previous work based on evaluations of chemical standards [16]. This result is primarily due to different retention mechanisms, chromatographic conditions, and above all ionization modes. 

Interestingly, the comparison of the AIF with IM ramped and fixed collision energies and AIF ramped and fixed collision energies affords complementary information for metabolite annotation when considering only RPLC and zHILIC (Supplementary Figures S4 and S8), while this was not the case for aHILIC (Supplementary Figure S7). By looking at the *m/z* vs. Rt maps, it is apparent that many annotated compounds coelute in aHILIC compared to RPLC and zHILIC (Supplementary Figures S9–S11). It can also be observed that some polar compounds are poorly retained in RPLC and elute at the dead time (Supplementary Figure S9). Therefore, alternative LC methods, such as aHILIC and zHILIC, showing complementary retention properties are needed for quantitative analysis by avoiding intense ion suppression at the dead time and achieving sufficient separation of the compounds for adequate MS analysis. Moreover, it is possible to identify these metabolites with greater confidence by reducing the risk of false annotations. For aHILIC, no complementarity was observed between AIF with IM and AIF ramp vs. fixed collision energies modalities because numerous metabolites coeluted in either the void volume or in specific regions at approximately 5 and 7 min of the *m/z* vs. Rt map (Supplementary Figure S10). Therefore, it is quite difficult to assess differences in the various tandem MS modalities with coeluting metabolites because the analytical time window is somewhat limited. Coeluting metabolites in 5 and 7 min regions correspond primarily to lipids (phosphatidylcholines) but also to amino acids.

To summarize, by selecting only the most informative tandem MS mode, i.e., AIF with and without IM using two ramps of 10–60 eV and 30–60 eV, a limited number of annotated metabolites will be lost. In fact, 290 metabolites could be found, corresponding to a loss of metabolite coverage of 14% (Figure 5b). This is extremely interesting because by using only 12 analytical runs (four for each chromatographic mode) instead of 30, a better analytical throughput is reached for an almost negligible loss of metabolite coverage. This result should be put in the context that complementarity in the metabolite annotation of diverse chemical classes gives no indication of whether the detected metabolites are analyzed in an optimal way, i.e., if they are sufficiently retained and with as little coelution of interfering compounds as possible. Furthermore, because it is not possible to predict what additional or common chemical information would be obtained using much larger MS/MS external libraries such as MoNA, we consider that three LC methods with four tandem MS acquisition modes using ramp collision energies, two using IM and two others without, represent a solid compromise for obtaining extensive metabolite coverage with Level 2 annotation.

## 3. Materials and Methods

### 3.1. Chemicals

UPLC-MS-grade acetonitrile (ACN), methanol (MeOH), isopropanol (iPrOH), and water (H_2_O) were obtained from Fisher (Fisher Scientific, Loughborough, UK). UPLC-MS-grade formic acid (FA) was supplied by Biosolve (Valkenswaard, Netherlands). MS-grade ammonium formate and ammonium hydroxide were obtained from Sigma-Aldrich (Buchs, Switzerland). The major mix IMS/ToF calibration kit and leucine-enkephalin were acquired from Waters (Wilmslow, UK).

### 3.2. Human Plasma

Six different heparinized plasma samples, which were obtained from healthy donors (three women and three men), were obtained from the Blood Transfusion Center (BTC) at Geneva University Hospitals (HUG).

Standard Reference Material for human plasma (SRM 1950) approved by the National Institute of Standards and Technology (NIST) was purchased from Sigma-Aldrich (Buchs, Switzerland). Good sample collection and storage practices were ensured [47].

### 3.3. Sample Preparation

Plasma samples from donors were used to condition the LC system and the MS source before analyzing the NIST plasma samples. The samples were collected, aliquoted, and stored at −80 °C. The aliquots were thawed on ice, and equal volumes of each donor were pooled. Protein precipitation was performed by adding 960 µL of cold MeOH (−20 °C) to 240 µL of pooled plasma. The samples were vortexed for 20 s, mixed at 1200 rpm for 30 min at 4 °C, and centrifuged at 14,000 g for 20 min at 4 °C. The supernatants of each sample were divided into three 300 µL aliquots. Each aliquot was evaporated to dryness (6 h) using a SpeedVac (ThermoFisher, Langenselbold, Germany) and stored dry at −80 °C. Before analysis, the samples were thawed on ice, reconstituted in 120 µL of ACN:H_2_O 50:50 *v/v*, and mixed at 1200 rpm for 15 min at 4 °C [20,21]. 

Sample preparation of the NIST plasma (SRM 1950) was performed by following the same protocol as for the pooled plasma depicted for healthy donor samples.

### 3.4. Liquid Chromatographic Conditions

For the UHPLC experiments, a Waters H-Class Acquity UPLC system (Waters, Milford, MA, USA) composed of a quaternary pump, an autosampler (for which the temperature was set at 7 °C) including a 15 µL flow-through-needle injector, and a two-way column manager was used. The injected volume was 10 µL.

RPLC separations were performed on a Kinetex C18 column (150 × 2.1 mm, 1.7 µm) and the corresponding SecurityGuard Ultra precolumn and holder (Phenomenex, Torrance, CA, USA). Solvent A was H_2_O, and solvent B was ACN, both of which contained 0.1% FA. The column temperature and flow rate were set to 30 °C and 300 µL min^−1^, respectively. The gradient elution was as follows: 2–100% B in 14 min, hold for 3 min, then back to 2% B for 0.1 min and re-equilibration for 7.9 min. Two HILIC chromatographic methods were employed. In the first one (aHILIC), separations were performed on an Acquity UPLC BEH amide column (150 × 2.1 mm, 1.7 µm) and the appropriate VanGuard^TM^ precolumn (Waters, Milford, MA, USA). Solvent A was ACN:H_2_O 95:5 *v/v* and solvent B was ACN:H_2_O 30:70 *v/v* containing 10 mM ammonium formate adjusted to pH 6.5 in H_2_O before being mixed with ACN. The column temperature and flow rate were set to 40 °C and 500 µL min^−1^, respectively. The gradient elution was as follows: 0% B for 2 min, increasing to 70% B during 18 min, holding for 3 min at 70% B and then returning back to 0% B in 1 min and re-equilibrating the column for 7 min. For the second one (zHILIC), separations were performed on a SeQuant® Zic-pHILIC column (150 × 2.1 mm, 5 µm) and the appropriate guard kit (Merck, Darmstadt, Germany). Solvent A was ACN and solvent B was H_2_O containing 2.8 mM ammonium formate adjusted to pH 9. The column temperature and flow rate were set to 40 °C and 300 µL min^−1^, respectively. The gradient elution was as follows: 5% B for 1 min, increasing to 51% B during 9 min, holding for 3 min at 51% B, and then returning back to 5% B in 0.1 min and re-equilibrating the column for 6.9 min. These analytical protocols were chosen based on previous articles [16,31].

### 3.5. Mass Spectrometric Conditions

The IM-HRMS experiments were performed on a Vion TWIMS-QToF mass spectrometer (Waters, Manchester, UK) equipped with an electrospray ionization (ESI) source. Analyses were performed in positive (for RPLC and aHILIC) and negative (for zHILIC) electrospray ionization (ESI) modes to acquire continuum data over a range of 50–1000 *m/z* with a scan time of 0.2 s. The source parameters were set as follows: the capillary and cone voltages were set to 0.6 kV for ESI+ and −2.0 kV for ESI− and 40 V for both ESI modes. The source and desolvation temperatures were set at 120 and 500 °C, cone and desolvation gas flows were 50 and 800 L/h, respectively. The velocity and height of StepWave1 and StepWave2 were set to 300 m/s and 5 V and to 200 m/s and 30 V, respectively.

As reported in Table 1, different tandem MS modalities were used with and without IM. The settings of the analyses performed using IM (so-called high definition modes, HDMS^E^ and HDDA) and without ion mobility (MS^E^ and DDA) consisted of trap wave velocities at 100 m/s and 450 m/s, respectively. Trap pulse height A was set to 15 V; trap pulse height B was 5 V; the IM wave velocity was 250 m/s (HDMS^E^ and HDDA) or 300 m/s (MS^E^ and DDA); the IM pulse heights were 45 V (HDMS^E^ and HDDA) and 15 V (MS^E^ and DDA); the wave delay was set at 20 pushes; and the gate delay was 0 ms. Gas flows of the ion mobility instrument were set to 1.60 L min^–1^ for trap gas and 25 mL min^–1^ for IM gas.

For the HDMS^E^ and MS^E^ acquisition modes, no particular parameters of the Vion had to be adjusted for tandem MS, since this mode performs all the ion fragmentation except that the acquisition time used here was the analysis method run time. For the HDDA and DDA acquisition modes, MS/MS acquisition was triggered when the intensity exceeded 2000 detector counts, a maximum of three simultaneous MS/MS acquisitions was set, MS/MS acquisition was performed until a timeout of 0.80 s, and the acquisition time corresponded to the analysis method run time. For the collision energy, 6.0 eV was used for the low energy, and the high energies were either ramped (from 10 to 60 eV or 30 to 60 eV) or fixed values (14, 28, or 56 eV).

Nitrogen was used as a collision gas. Leucine-enkephalin served as a lock mass (*m/z* 556.2771 for positive mode and *m/z* 554.2615 for negative mode) and was infused at 5 min intervals. The ^TW^CCS_N2_ and mass calibration of the instrument were performed using the major mix IMS/ToF calibration kit (Waters, Wilmslow, UK). Waters UNIFI v1.9.3 software was used for instrument control and data acquisition.

### 3.6. Exclusion Lists

For the HDDA and DDA acquisition modes, all the ions that were fragmented in the first analysis were excluded from a second analytical run using the following criteria: retention time tolerance of 30 s (for RPLC) or 45 s (for both HILIC methods), mass tolerance of 5 mDa on the precursor ion, 7 Å^2^ tolerance for the measured ^TW^CCS_N2_ (when ion mobility was used) and a MS/MS dynamic peak exclusion with durations of 10 s (RPLC) and 20 s (for both HILIC methods). The lists of fragmented ions were generated in UNIFI by applying two processing methods for the HDDA and DDA acquisition modes, respectively. The following criteria were used for DDA and HDDA: MS and MS/MS ion intensity threshold: 100 and 30 counts, respectively, maximum number of MS and MS/MS ions to keep per set mass: 2000, and maximum allowed number of peaks per chromatogram: 20,000. Then, a CSV file was generated for each analytical run containing the detected *m/z*, ^TW^CCS_N2_, and retention times as well as the exclusion criteria for all the precursors. Lastly, the CSV files were imported in UNIFI to exclude the fragmented ions from the second analytical run.

### 3.7. Analytical Sequence

The same analytical sequences were used for all three chromatographic modes, i.e., RPLC, aHILIC, and zHILIC. The initial analytical sequence contained all the MS/MS acquisition modes investigated here, and the second analytical sequence contained only HDDA and DDA acquisition modes with exclusion lists. Details on the analytical sequences can be found in Supplementary Tables S1 and S2.

To evaluate all tandem MS/MS modes as fairly and repeatably as possible, conditioning samples were included at the beginning of the analytical sequence. These conditioning runs included blank (i), system suitability test (SST) (ii), human plasma (iii), and NIST plasma (iv) samples with the following goals:

i. Condition the LC to the gradient mode.
ii. Evaluate the performance of the analytical platform [31].
iii. Condition and stabilize the LC and HRMS systems to the plasma matrix.
iv. Use the exact same matrix sample as the following runs of interest.

### 3.8. Analysis of Raw Data and Metabolite Annotation

Data processing was performed using Progenesis QI v2.3 (Nonlinear Dynamics, Waters, Newcastle upon Tyne, UK). Peak picking, adduct deconvolution, and feature annotation were performed sequentially in the software as described in [31]. 

The following tolerances were used for metabolite annotation using the built-in Waters Metabolic Profiling CCS Library: 5 ppm for precursor and fragment mass, 2.5% in the case of ^TW^CCS_N2_ and an isotopic similarity > 60%. MS/MS fragmentation matches between the evaluated and reference spectra were calculated based on the cosine similarity method implemented in the software. This algorithm is also used by MassBank [52,53]. The cosine similarity method rates fragmentation scores between 0 (worst case) and 100 (best case). Obtained scores will depend on the matching of the evaluated MS/MS spectrum with the reference one. The presence of non-matching peaks or the absence of peaks in the evaluated MS/MS spectrum compared to the reference one will lower the obtained score. Finally, for a given metabolite identification, the software evaluates up to five different properties of each feature that will contribute to its overall score, i.e., mass, isotope distribution, retention time, CCS, and fragmentation score. Each one of these individual scores takes a value between 0 and 100. If a certain property of a feature does not match the reference data, the related score is 0. The overall score is the mean of these five scores.

## 4. Conclusions

The performance of different tandem MS acquisition modes with and without IM on a QToF platform were studied for efficient and broad metabolite annotation in human plasma in an untargeted metabolomics context. We first assessed the DDA and AIF approaches with and without IM using a RPLC chromatographic mode and annotated 186 metabolites either by accurate mass-^TW^CCS_N2_-MS/MS, accurate mass-^TW^CCS_N2_, or accurate mass-MS/MS. 

Based on the generated data, we have determined that the DDA acquisition modalities: (1) did not bring a significant additional amount of chemical coverage, (2) were more time-consuming with two analytical sequences (the second one had an exclusion list of all the fragmented ions from the first one) for reaching comparable annotated metabolites, and (3) cleaner MS/MS spectra can be obtained using IM performing AIF acquisitions (i.e., HDMS^E^), as highlighted for tryptophan. 

In fact, AIF without IM was able to annotate the largest number of metabolites, a significant proportion of which have only been detected using this modality. Therefore, we chose to investigate the AIF acquisition modes with and without IM for the aHILIC and zHILIC chromatographic modes. These two additional chromatographic modes were selected due to their complementary retention mechanisms and their ability to analyze a wide chemical range of polar metabolites. Totals of 187 and 177 unique metabolites were annotated, either by accurate mass-^TW^CCS_N2_-MS/MS, accurate mass-^TW^CCS_N2_, or accurate mass-MS/MS, depending on the acquisition mode specifics.

We highlighted that more metabolites are annotated when IM is off and that ramped CID acquisition modes provided similar performance (total metabolites annotated) compared to traditional fixed CID at low, medium, and high energies. Therefore, to save time and the volume of valuable biological samples, we recommend the use of only one or two MS/MS ramp acquisitions with and without IM. However, the resolution of the IM cell used in this study is limited when compared to other IM-HRMS instruments available on the market, and thus, these results could be different when using other MS instruments that provide higher IM resolution [54].

By merging data from the three chromatographic modes, 338 unique metabolites were annotated using all the investigated AIF acquisition modes. We showed that zHILIC is highly complementary to RPLC and/or aHILIC in terms of metabolome coverage. By selecting the most appropriate AIF acquisition modes to conduct untargeted metabolomics studies, i.e., AIF with and without IM using ramps of 10–60 and 30–60 eV, 290 unique metabolites could be found, highlighting that complementary acquisition modes are needed to reach broader metabolome coverage, i.e., by using AIF with IM (HDMS^E^), for instance.

Finally, we believe that continuing the work on integrating all the MS/MS information from different publicly available databases, as initiated by Blaženović et al. and Gil-de-la-Fuente et al., will provide access to a more extensive chemical knowledge of the biological samples analysed by the scientific community [22,30] and would be highly beneficial. The present article only presents an evaluation of different tandem MS acquisition modes and allows researchers to choose the most appropriate one to obtain the widest metabolite coverage possible for future metabolomics experiments.

## Figures and Tables

**Table 1:** All tandem MS acquisition modes used with and without ion mobility spectrometry (IM).

Acquisition Mode	AIF with IM	DDA with IM	AIF without IM	DDA without IM
Ramped	IM-AIF Ramp 10–60	IM-DDA Ramp 10–60	AIF Ramp 10–60	DDA Ramp 10–60
	IM-AIF Ramp 30–60	IM-DDA Ramp 30–60	AIF Ramp 30–60	DDA Ramp 30–60
Fixed	IM-AIF Fixed 14	IM-DDA Fixed 14	AIF Fixed 14	DDA Fixed 14
	IM-AIF Fixed 28	IM-DDA Fixed 28	AIF Fixed 28	DDA Fixed 28
	IM-AIF Fixed 56	IM-DDA Fixed 56	AIF Fixed 56	DDA Fixed 56

## Supplementary Materials

The following are available online at https://www.mdpi.com/2218-1989/10/11/464/s1, Table S1: Initial analytical sequence. Table S2: Analytical sequence with exclusion lists. Figure S1: Evaluation of the proposed annotations for each annotated metabolite by PQI in the corresponding filtered features list for RPLC ESI+. Figure S2: Chemical classes of the 55 annotated metabolites with each of IM-AIF, IM-DDA, AIF and DDA acquisition modes for RPLC ESI+. Figure S3: Chemical classes of the 42 annotated metabolites by AIF acquisition modes only for RPLC ESI+. Figure S4: Annotated metabolites by IM-AIF and AIF ramped vs. fixed dissociation energies for RPLC ESI+. Figure S5: Overall non-ambiguous metabolite annotations made by accurate mass-^TW^CCS_N2_-MS/MS (blue), accurate mass-^TW^CCS_N2_ (red) and accurate mass-MS/MS (green) for aHILIC ESI+. Figure S6: Overall non-ambiguous metabolite annotations made by accurate mass-^TW^CCS_N2_-MS/MS (blue), accurate mass-^TW^CCS_N2_ (red) and accurate mass-MS/MS (green) for zHILIC ESI-. Figure S7: Annotated metabolites by IM-AIF and AIF ramped vs. fixed dissociation energies for aHILIC ESI+. Figure S8: Annotated metabolites by IM-AIF and AIF ramped vs. fixed dissociation energies for zHILIC ESI-. Figure S9: *m/z* vs- Rt map of annotated metabolites for all IM-AIF (a) and AIF (b) acquisition modes in RPLC ESI+. Blue and orange dots are metabolites annotated by ramped and fixed collision energies modalities, respectively. Figure S10: *m/z* vs- Rt map of annotated metabolites for all IM-AIF (a) and AIF (b) acquisition modes in aHILIC ESI+. Blue and orange dots are metabolites annotated by ramped and fixed collision energies modalities, respectively. Figure S11: *m/z* vs- Rt map of annotated metabolites for all IM-AIF (a) and AIF (b) acquisition modes in zHILIC ESI-. Blue and orange dots are metabolites annotated by ramped and fixed collision energies modalities, respectively. Tables S3, S4 and S5: List of annotated metabolites for each LC technique (i.e., for RPLC ESI+, aHILIC ESI+ and zHILIC ESI-, respectively) and tandem MS modality, along with various information comprising the PubChem ID and name, Rt, *m/z*, ^TW^CCS_N2_ (when available), chemical class, MS/MS mode(s), overall score, fragmentation score, mass error (ppm), isotope similarity and delta CCS.
