# Supplementary material for: Evaluation of Different Tandem MS Acquisition Modes to Support Metabolite Annotation in Human Plasma Using Ultra High-Performance Liquid Chromatography High-Resolution Mass Spectrometry for Untargeted Metabolomics
Source: Metabolites. 2020 Nov 15;10(11):464. doi: 10.3390/metabo10110464 (PMC7697060; doi:10.3390/metabo10110464)
Supplement: Supplementary file 1 [file metabolites-10-00464-s001.zip › Supplementary Material/Figure S1.pptx]

## Slide 1
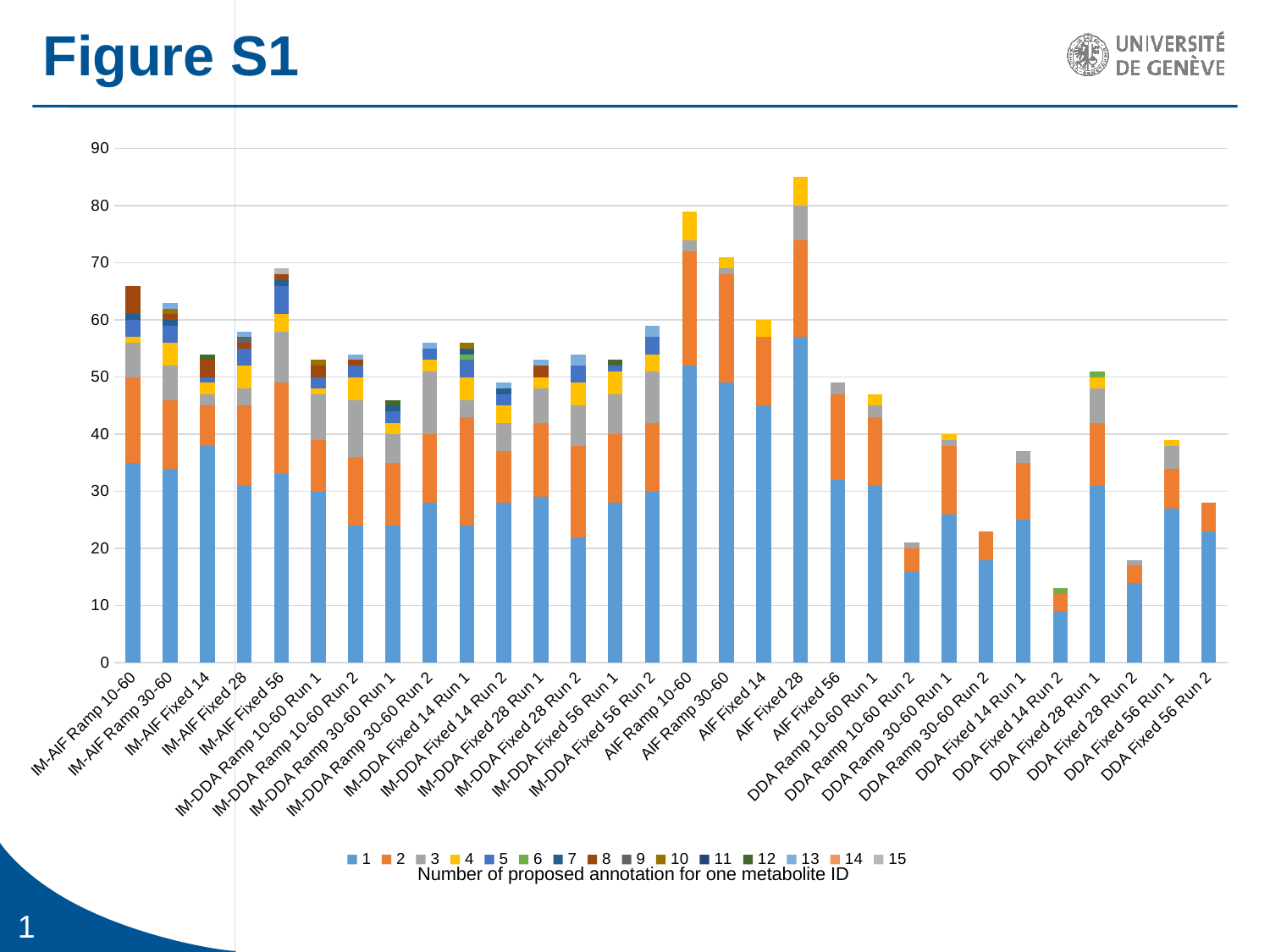

Figure S1
### Chart
| Category | 1 | 2 | 3 | 4 | 5 | 6 | 7 | 8 | 9 | 10 | 11 | 12 | 13 | 14 | 15 |
|---|---|---|---|---|---|---|---|---|---|---|---|---|---|---|---|
| IM-AIF Ramp 10-60 | 35.0 | 15.0 | 6.0 | 1.0 | 3.0 | 0.0 | 1.0 | 5.0 | 0.0 | 0.0 | 0.0 | 0.0 | 0.0 | 0.0 | 0.0 |
| IM-AIF Ramp 30-60 | 34.0 | 12.0 | 6.0 | 4.0 | 3.0 | 0.0 | 1.0 | 1.0 | 0.0 | 1.0 | 0.0 | 0.0 | 1.0 | 0.0 | 0.0 |
| IM-AIF Fixed 14 | 38.0 | 7.0 | 2.0 | 2.0 | 1.0 | 0.0 | 0.0 | 3.0 | 0.0 | 0.0 | 0.0 | 1.0 | 0.0 | 0.0 | 0.0 |
| IM-AIF Fixed 28 | 31.0 | 14.0 | 3.0 | 4.0 | 3.0 | 0.0 | 0.0 | 1.0 | 1.0 | 0.0 | 0.0 | 0.0 | 1.0 | 0.0 | 0.0 |
| IM-AIF Fixed 56 | 33.0 | 16.0 | 9.0 | 3.0 | 5.0 | 0.0 | 1.0 | 1.0 | 0.0 | 0.0 | 0.0 | 0.0 | 0.0 | 0.0 | 1.0 |
| IM-DDA Ramp 10-60 Run 1 | 30.0 | 9.0 | 8.0 | 1.0 | 2.0 | 0.0 | 0.0 | 2.0 | 0.0 | 1.0 | 0.0 | 0.0 | 0.0 | 0.0 | 0.0 |
| IM-DDA Ramp 10-60 Run 2 | 24.0 | 12.0 | 10.0 | 4.0 | 2.0 | 0.0 | 0.0 | 1.0 | 0.0 | 0.0 | 0.0 | 0.0 | 1.0 | 0.0 | 0.0 |
| IM-DDA Ramp 30-60 Run 1 | 24.0 | 11.0 | 5.0 | 2.0 | 2.0 | 0.0 | 1.0 | 0.0 | 0.0 | 0.0 | 0.0 | 1.0 | 0.0 | 0.0 | 0.0 |
| IM-DDA Ramp 30-60 Run 2 | 28.0 | 12.0 | 11.0 | 2.0 | 2.0 | 0.0 | 0.0 | 0.0 | 0.0 | 0.0 | 0.0 | 0.0 | 1.0 | 0.0 | 0.0 |
| IM-DDA Fixed 14 Run 1 | 24.0 | 19.0 | 3.0 | 4.0 | 3.0 | 1.0 | 1.0 | 0.0 | 0.0 | 1.0 | 0.0 | 0.0 | 0.0 | 0.0 | 0.0 |
| IM-DDA Fixed 14 Run 2 | 28.0 | 9.0 | 5.0 | 3.0 | 2.0 | 0.0 | 1.0 | 0.0 | 0.0 | 0.0 | 0.0 | 0.0 | 1.0 | 0.0 | 0.0 |
| IM-DDA Fixed 28 Run 1 | 29.0 | 13.0 | 6.0 | 2.0 | 0.0 | 0.0 | 0.0 | 2.0 | 0.0 | 0.0 | 0.0 | 0.0 | 1.0 | 0.0 | 0.0 |
| IM-DDA Fixed 28 Run 2 | 22.0 | 16.0 | 7.0 | 4.0 | 3.0 | 0.0 | 0.0 | 0.0 | 0.0 | 0.0 | 0.0 | 0.0 | 2.0 | 0.0 | 0.0 |
| IM-DDA Fixed 56 Run 1 | 28.0 | 12.0 | 7.0 | 4.0 | 1.0 | 0.0 | 0.0 | 0.0 | 0.0 | 0.0 | 0.0 | 1.0 | 0.0 | 0.0 | 0.0 |
| IM-DDA Fixed 56 Run 2 | 30.0 | 12.0 | 9.0 | 3.0 | 3.0 | 0.0 | 0.0 | 0.0 | 0.0 | 0.0 | 0.0 | 0.0 | 2.0 | 0.0 | 0.0 |
| AIF Ramp 10-60 | 52.0 | 20.0 | 2.0 | 5.0 | 0.0 | 0.0 | 0.0 | 0.0 | 0.0 | 0.0 | 0.0 | 0.0 | 0.0 | 0.0 | 0.0 |
| AIF Ramp 30-60 | 49.0 | 19.0 | 1.0 | 2.0 | 0.0 | 0.0 | 0.0 | 0.0 | 0.0 | 0.0 | 0.0 | 0.0 | 0.0 | 0.0 | 0.0 |
| AIF Fixed 14 | 45.0 | 12.0 | 0.0 | 3.0 | 0.0 | 0.0 | 0.0 | 0.0 | 0.0 | 0.0 | 0.0 | 0.0 | 0.0 | 0.0 | 0.0 |
| AIF Fixed 28 | 57.0 | 17.0 | 6.0 | 5.0 | 0.0 | 0.0 | 0.0 | 0.0 | 0.0 | 0.0 | 0.0 | 0.0 | 0.0 | 0.0 | 0.0 |
| AIF Fixed 56 | 32.0 | 15.0 | 2.0 | 0.0 | 0.0 | 0.0 | 0.0 | 0.0 | 0.0 | 0.0 | 0.0 | 0.0 | 0.0 | 0.0 | 0.0 |
| DDA Ramp 10-60 Run 1 | 31.0 | 12.0 | 2.0 | 2.0 | 0.0 | 0.0 | 0.0 | 0.0 | 0.0 | 0.0 | 0.0 | 0.0 | 0.0 | 0.0 | 0.0 |
| DDA Ramp 10-60 Run 2 | 16.0 | 4.0 | 1.0 | 0.0 | 0.0 | 0.0 | 0.0 | 0.0 | 0.0 | 0.0 | 0.0 | 0.0 | 0.0 | 0.0 | 0.0 |
| DDA Ramp 30-60 Run 1 | 26.0 | 12.0 | 1.0 | 1.0 | 0.0 | 0.0 | 0.0 | 0.0 | 0.0 | 0.0 | 0.0 | 0.0 | 0.0 | 0.0 | 0.0 |
| DDA Ramp 30-60 Run 2 | 18.0 | 5.0 | 0.0 | 0.0 | 0.0 | 0.0 | 0.0 | 0.0 | 0.0 | 0.0 | 0.0 | 0.0 | 0.0 | 0.0 | 0.0 |
| DDA Fixed 14 Run 1 | 25.0 | 10.0 | 2.0 | 0.0 | 0.0 | 0.0 | 0.0 | 0.0 | 0.0 | 0.0 | 0.0 | 0.0 | 0.0 | 0.0 | 0.0 |
| DDA Fixed 14 Run 2 | 9.0 | 3.0 | 0.0 | 0.0 | 0.0 | 1.0 | 0.0 | 0.0 | 0.0 | 0.0 | 0.0 | 0.0 | 0.0 | 0.0 | 0.0 |
| DDA Fixed 28 Run 1 | 31.0 | 11.0 | 6.0 | 2.0 | 0.0 | 1.0 | 0.0 | 0.0 | 0.0 | 0.0 | 0.0 | 0.0 | 0.0 | 0.0 | 0.0 |
| DDA Fixed 28 Run 2 | 14.0 | 3.0 | 1.0 | 0.0 | 0.0 | 0.0 | 0.0 | 0.0 | 0.0 | 0.0 | 0.0 | 0.0 | 0.0 | 0.0 | 0.0 |
| DDA Fixed 56 Run 1 | 27.0 | 7.0 | 4.0 | 1.0 | 0.0 | 0.0 | 0.0 | 0.0 | 0.0 | 0.0 | 0.0 | 0.0 | 0.0 | 0.0 | 0.0 |
| DDA Fixed 56 Run 2 | 23.0 | 5.0 | 0.0 | 0.0 | 0.0 | 0.0 | 0.0 | 0.0 | 0.0 | 0.0 | 0.0 | 0.0 | 0.0 | 0.0 | 0.0 |Number of proposed annotation for one metabolite ID
