# Supplementary material for: Evaluation of Different Tandem MS Acquisition Modes to Support Metabolite Annotation in Human Plasma Using Ultra High-Performance Liquid Chromatography High-Resolution Mass Spectrometry for Untargeted Metabolomics
Source: Metabolites. 2020 Nov 15;10(11):464. doi: 10.3390/metabo10110464 (PMC7697060; doi:10.3390/metabo10110464)
Supplement: Supplementary file 1 [file metabolites-10-00464-s001.zip › Supplementary Material/Figure S2.pptx]

## Slide 1
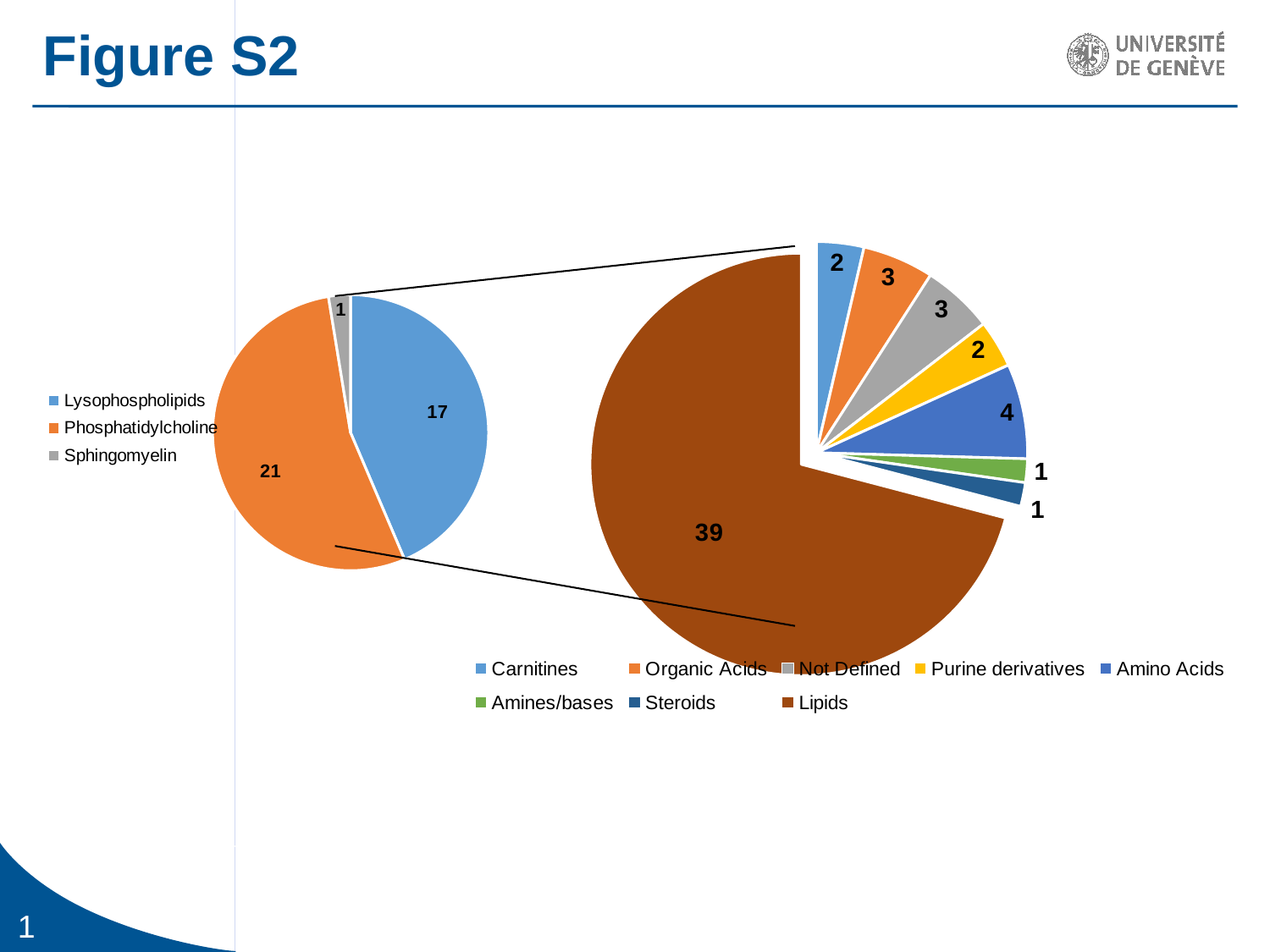

Figure S2
### Chart
| Category | |
|---|---|
| Carnitines | 2.0 |
| Organic Acids | 3.0 |
| Not Defined | 3.0 |
| Purine derivatives | 2.0 |
| Amino Acids | 4.0 |
| Amines/bases | 1.0 |
| Steroids | 1.0 |
| Lipids | 39.0 |
### Chart
| Category | |
|---|---|
| Lysophospholipids | 17.0 |
| Phosphatidylcholine | 21.0 |
| Sphingomyelin | 1.0 |
