# Supplementary material for: Evaluation of Different Tandem MS Acquisition Modes to Support Metabolite Annotation in Human Plasma Using Ultra High-Performance Liquid Chromatography High-Resolution Mass Spectrometry for Untargeted Metabolomics
Source: Metabolites. 2020 Nov 15;10(11):464. doi: 10.3390/metabo10110464 (PMC7697060; doi:10.3390/metabo10110464)
Supplement: Supplementary file 1 [file metabolites-10-00464-s001.zip › Supplementary Material/Tables S1 and S2.docx]

# Table S1: Initial analytical sequence

| **Samples Conditions** | **Location** | **Inj N°** | **NB** |
| --- | --- | --- | --- |
| **Blanks** | 1A1 | 1 | 7 |
| **Mix SST** | 1A2 | 8 | 3 |
| **Plasma Trash 1** | 1A3 | 11 | 5 |
| **Plasma Trash 2** | 1A4 | 16 | 5 |
| **Plasma NIST Trash** | 1A5 | 21 | 3 |
| NIST AIF Fixed 28eV | 1B1 | 24 | 1 |
| NIST IM-DDA Fixed 56eV | 1B1 | 25 | 1 |
| NIST IM-AIF Ramp 30-60eV | 1B1 | 26 | 1 |
| NIST DDA Fixed 56eV | 1B1 | 27 | 1 |
| NIST IM-DDA Ramp 30-60eV | 1B1 | 28 | 1 |
| NIST AIF Fixed 14eV | 1B2 | 29 | 1 |
| NIST IM-AIF Fixed 28eV | 1B2 | 30 | 1 |
| NIST DDA Ramp 10-60eV | 1B2 | 31 | 1 |
| NIST IM-DDA Fixed 28eV | 1B2 | 32 | 1 |
| NIST AIF Ramp 10-60eV | 1B2 | 33 | 1 |
| NIST DDA Ramp 30-60eV | 1B3 | 34 | 1 |
| NIST IM-AIF Fixed 56eV | 1B3 | 35 | 1 |
| NIST IM-DDA Ramp 10-60eV | 1B3 | 36 | 1 |
| NIST AIF Fixed 56eV | 1B3 | 37 | 1 |
| NIST IM-DDA Fixed 14eV | 1B3 | 38 | 1 |
| NIST DDA Fixed 14eV | 1B4 | 39 | 1 |
| NIST IM-AIF Ramp 10-60eV | 1B4 | 40 | 1 |
| NIST AIF Ramp 30-60eV | 1B4 | 41 | 1 |
| NIST DDA Fixed 28eV | 1B4 | 42 | 1 |
| NIST IM-AIF Fixed 14eV | 1B4 | 43 | 1 |
| **Mix SST** | 1A2 | 44 | 1 |
| **STOP** | 1A1 | 45 | 1 |

# Table S2: Analytical sequence with exclusion lists

| **Samples Conditions** | **Location** | **Inj N°** | **NB** |
| --- | --- | --- | --- |
| **Blanks** | 1A1 | 1 | 7 |
| **Mix SST** | 1A2 | 8 | 3 |
| **Plasma Trash 1** | 1A3 | 11 | 5 |
| **Plasma Trash 2** | 1A4 | 16 | 5 |
| **Plasma NIST Trash** | 1A5 | 21 | 3 |
| NIST DDA Ramp 10-60eV | 1B1 | 24 | 1 |
| NIST IM-DDA Ramp 30-60eV | 1B1 | 25 | 1 |
| NIST DDA Fixed 14eV | 1B1 | 26 | 1 |
| NIST IM-DDA Fixed 56eV | 1B1 | 27 | 1 |
| NIST DDA Fixed 28eV | 1B1 | 28 | 1 |
| NIST IM-DDA Ramp 10-60eV | 1B2 | 29 | 1 |
| NIST DDA Ramp 30-60eV | 1B2 | 30 | 1 |
| NIST IM-DDA Fixed 14eV | 1B2 | 31 | 1 |
| NIST DDA Fixed 56eV | 1B2 | 32 | 1 |
| NIST IM-DDA Fixed 28eV | 1B2 | 33 | 1 |
| **Mix SST** | 1A2 | 34 | 1 |
| **STOP** | 1A1 | 35 | 1 |
