# Supplementary figures and images for: Evaluation of Different Tandem MS Acquisition Modes to Support Metabolite Annotation in Human Plasma Using Ultra High-Performance Liquid Chromatography High-Resolution Mass Spectrometry for Untargeted Metabolomics
Source: Metabolites. 2020 Nov 15;10(11):464. doi: 10.3390/metabo10110464 (PMC7697060; doi:10.3390/metabo10110464)

## Slide 1
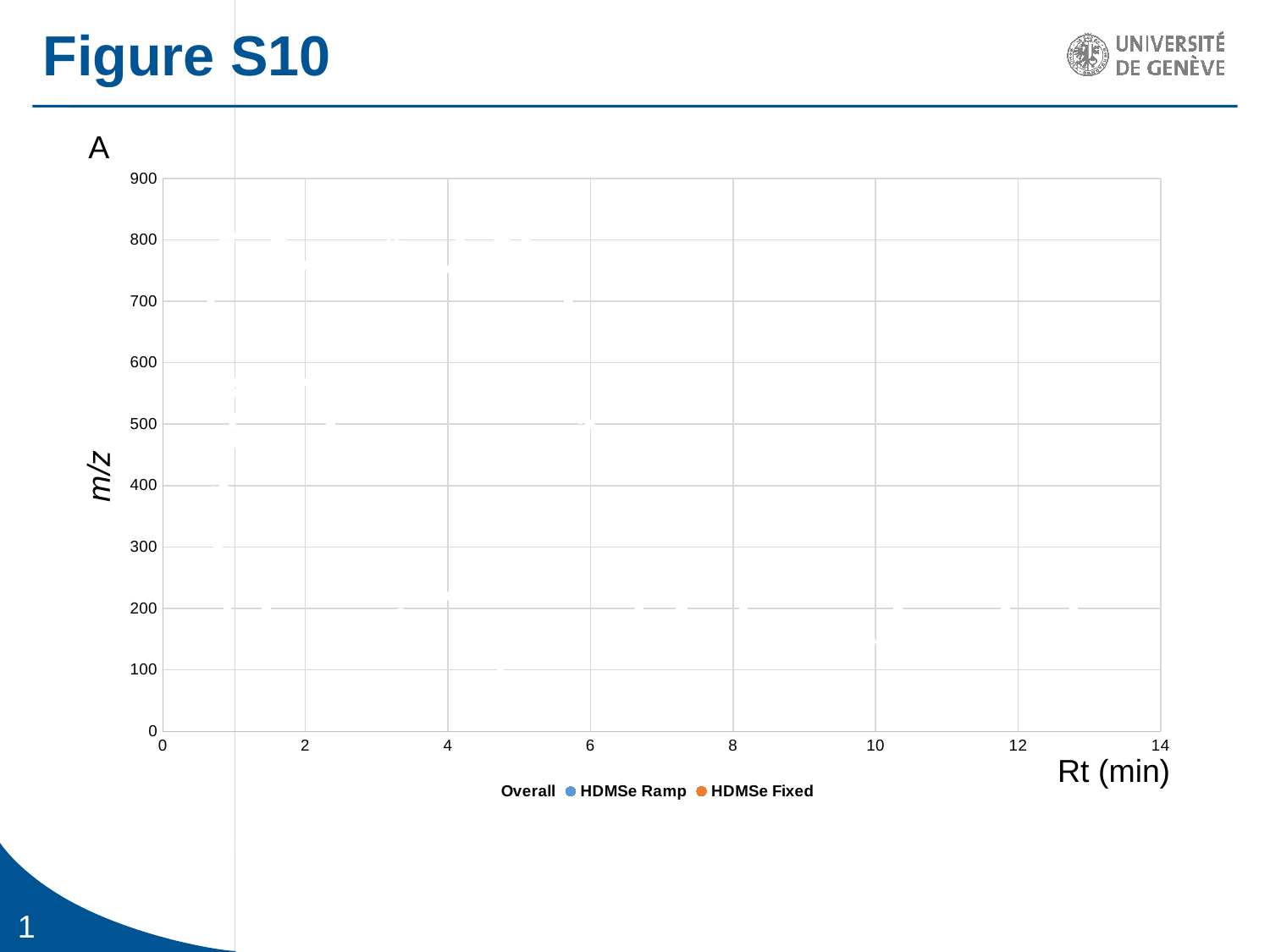

Figure S10
A
### Chart
| Category | | | |
|---|---|---|---|m/z
Rt (min)

## Slide 2
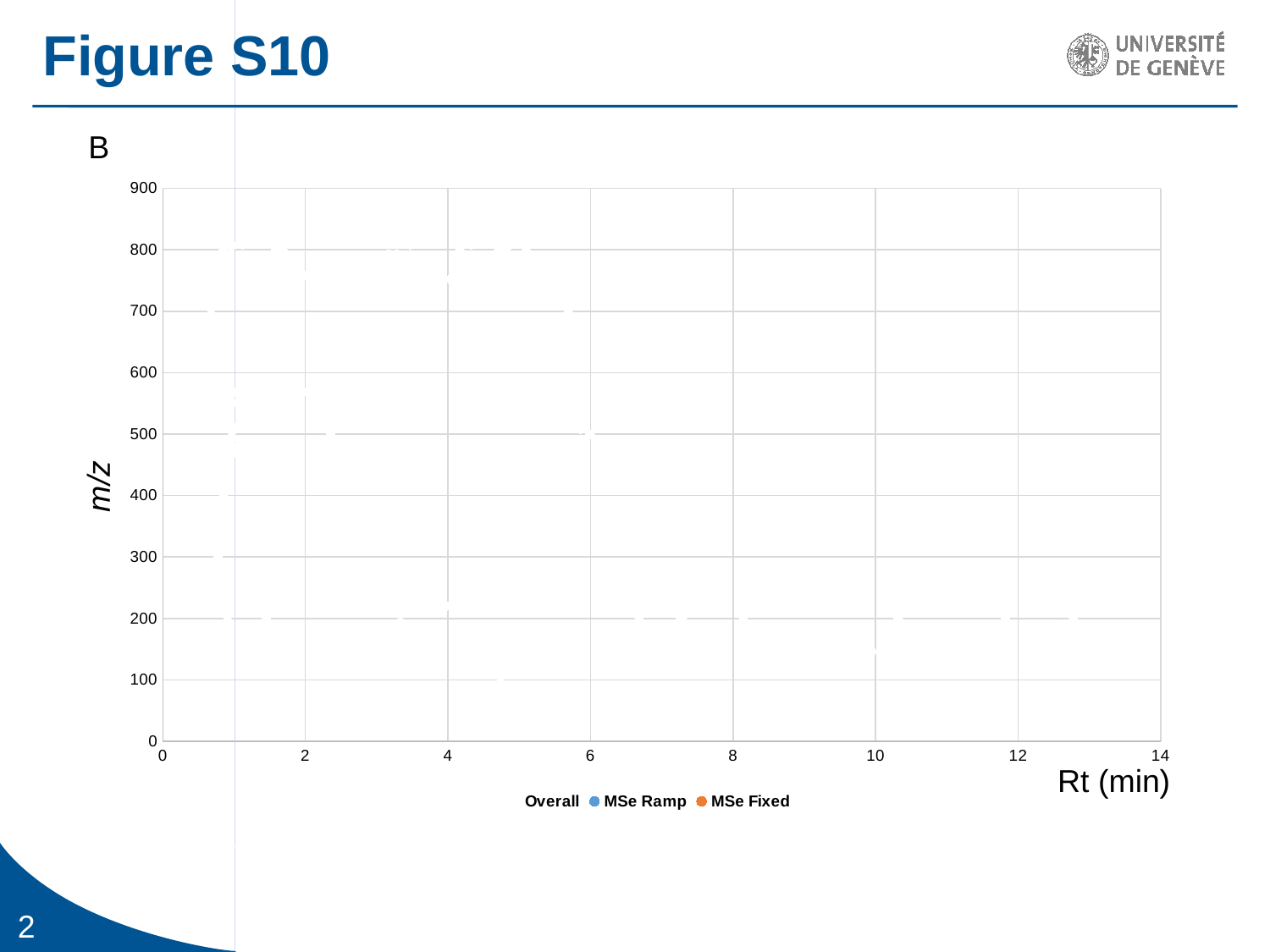

Figure S10
B
### Chart
| Category | | | |
|---|---|---|---|m/z
Rt (min)

Supplement: Supplementary file 1 [file metabolites-10-00464-s001.zip › Supplementary Material/Figure S10.pptx]

## Slide 1
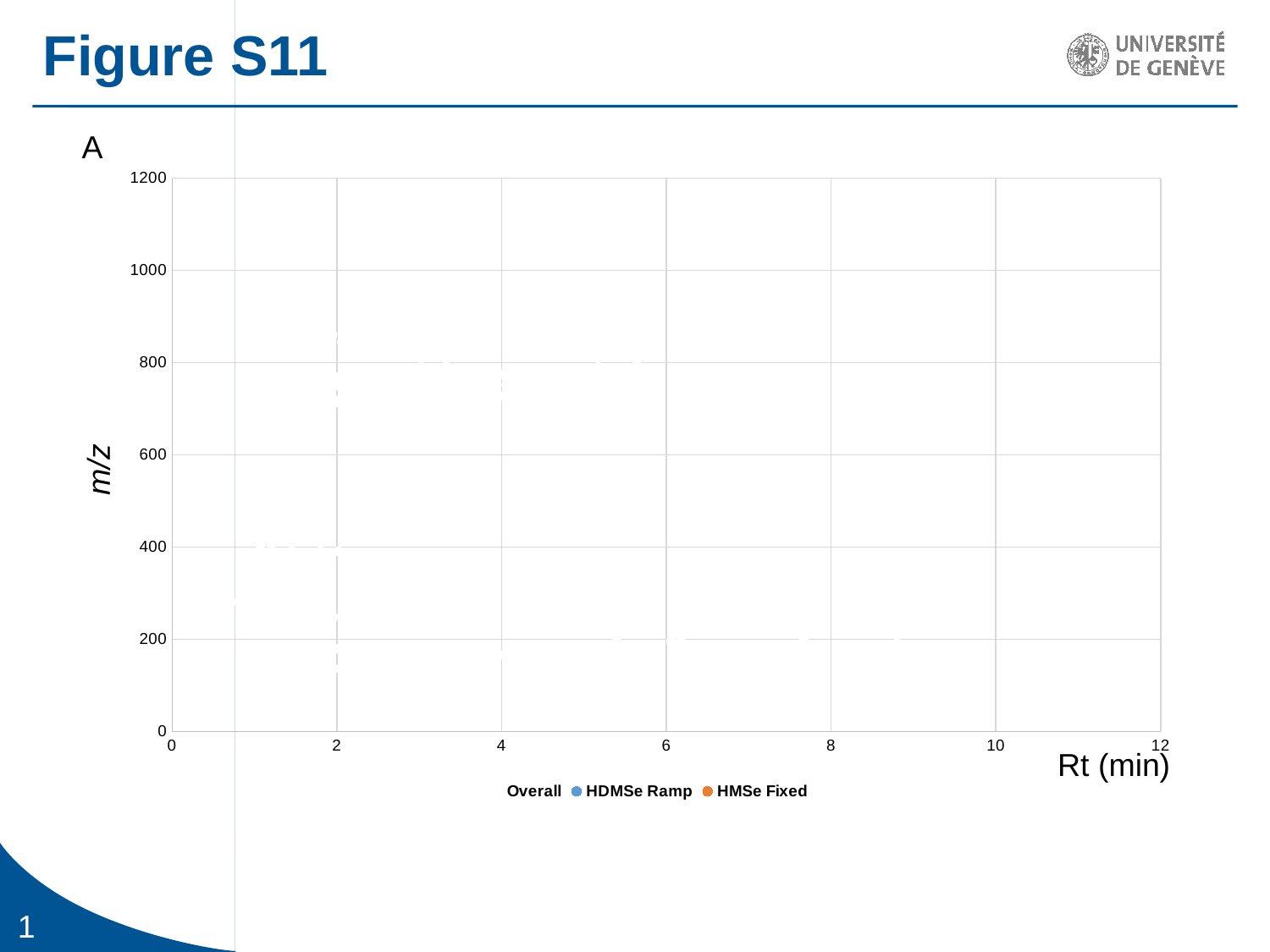

Figure S11
A
### Chart
| Category | | | |
|---|---|---|---|m/z
Rt (min)

## Slide 2
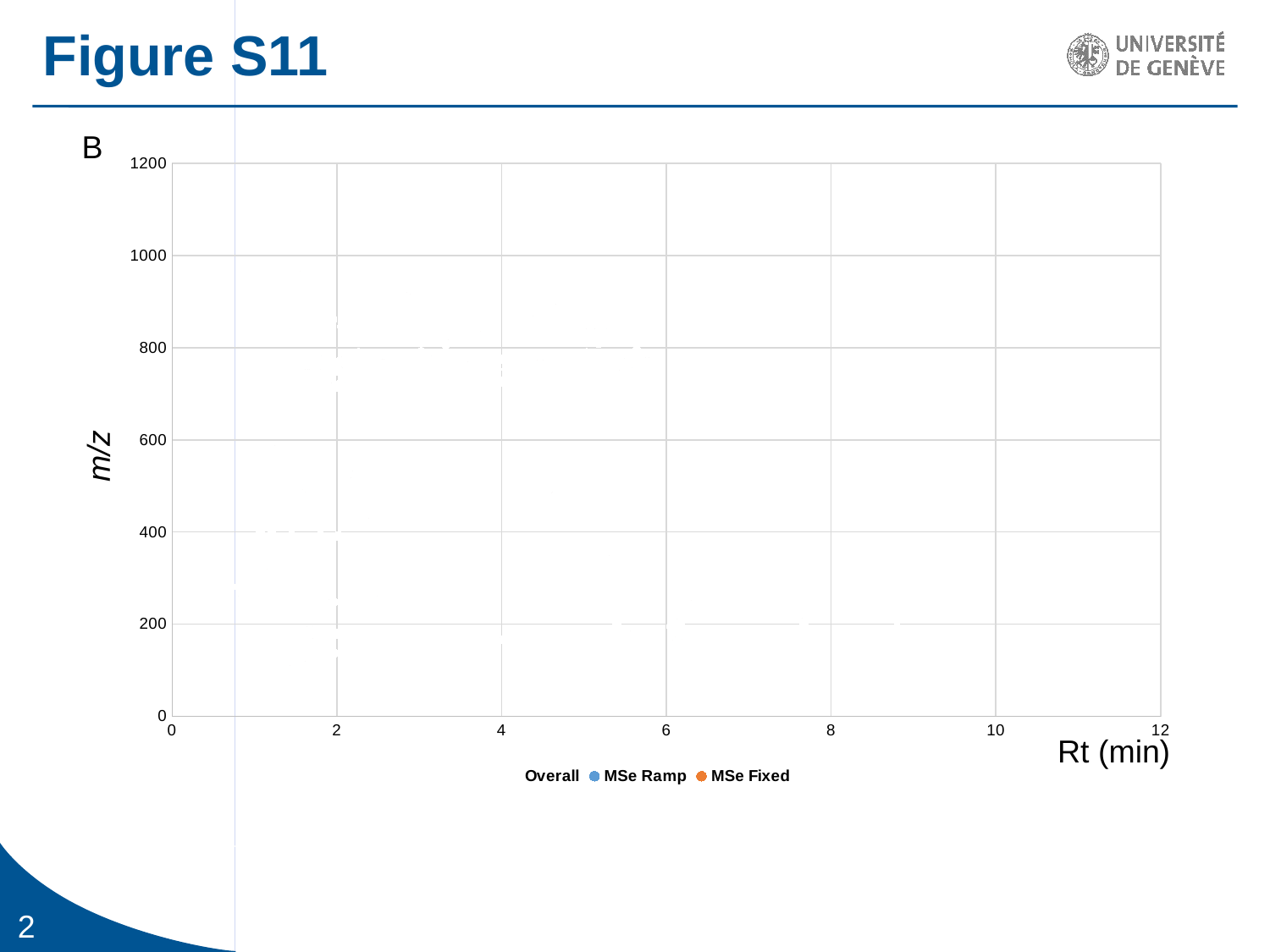

Figure S11
B
### Chart
| Category | | | |
|---|---|---|---|m/z
Rt (min)

Supplement: Supplementary file 1 [file metabolites-10-00464-s001.zip › Supplementary Material/Figure S11.pptx]

## Slide 1
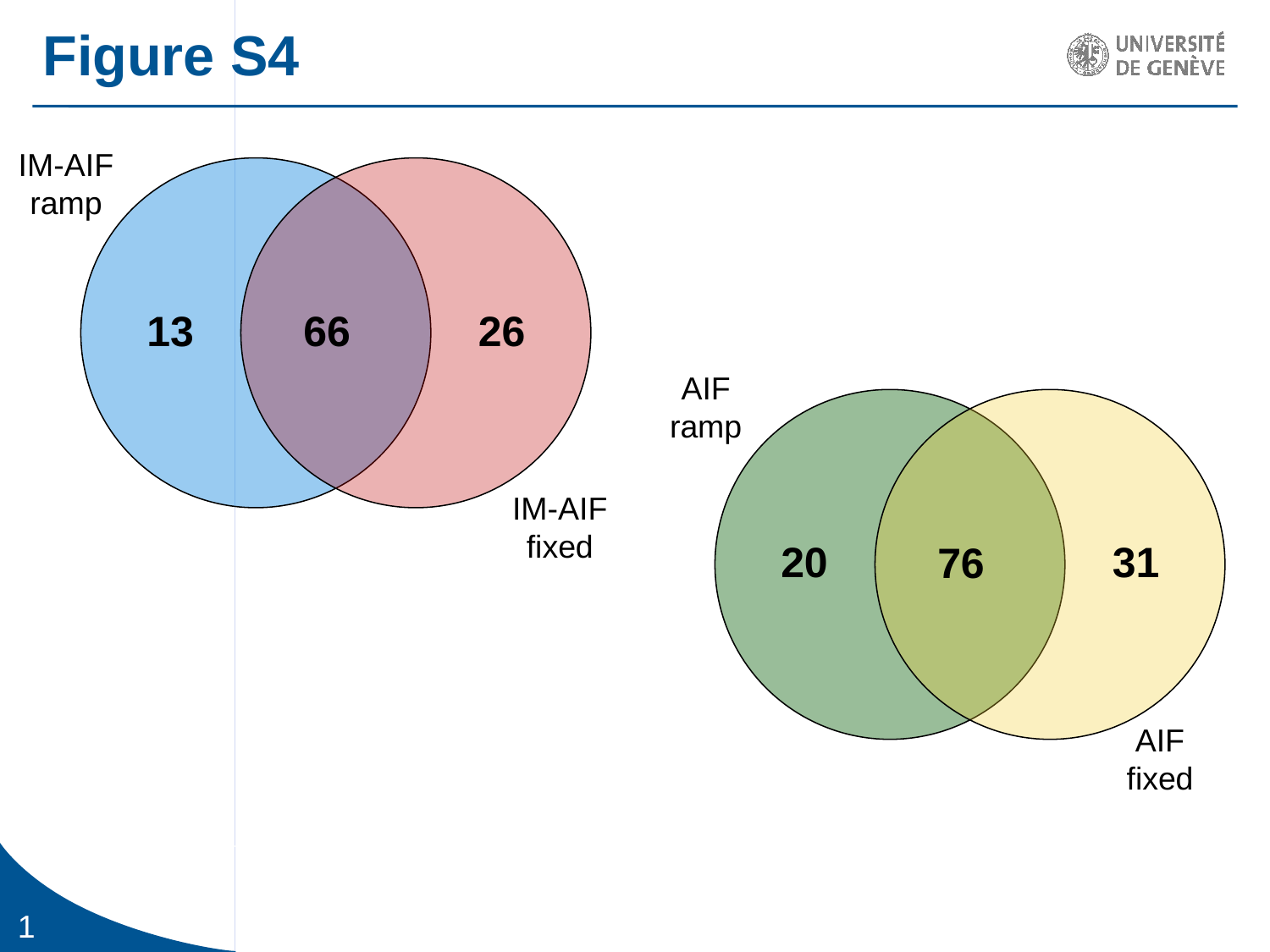

Figure S4
IM-AIF
ramp
13
26
66
AIF
ramp
IM-AIF
fixed
20
31
76
AIF
fixed

Supplement: Supplementary file 1 [file metabolites-10-00464-s001.zip › Supplementary Material/Figure S4.pptx]

## Slide 1
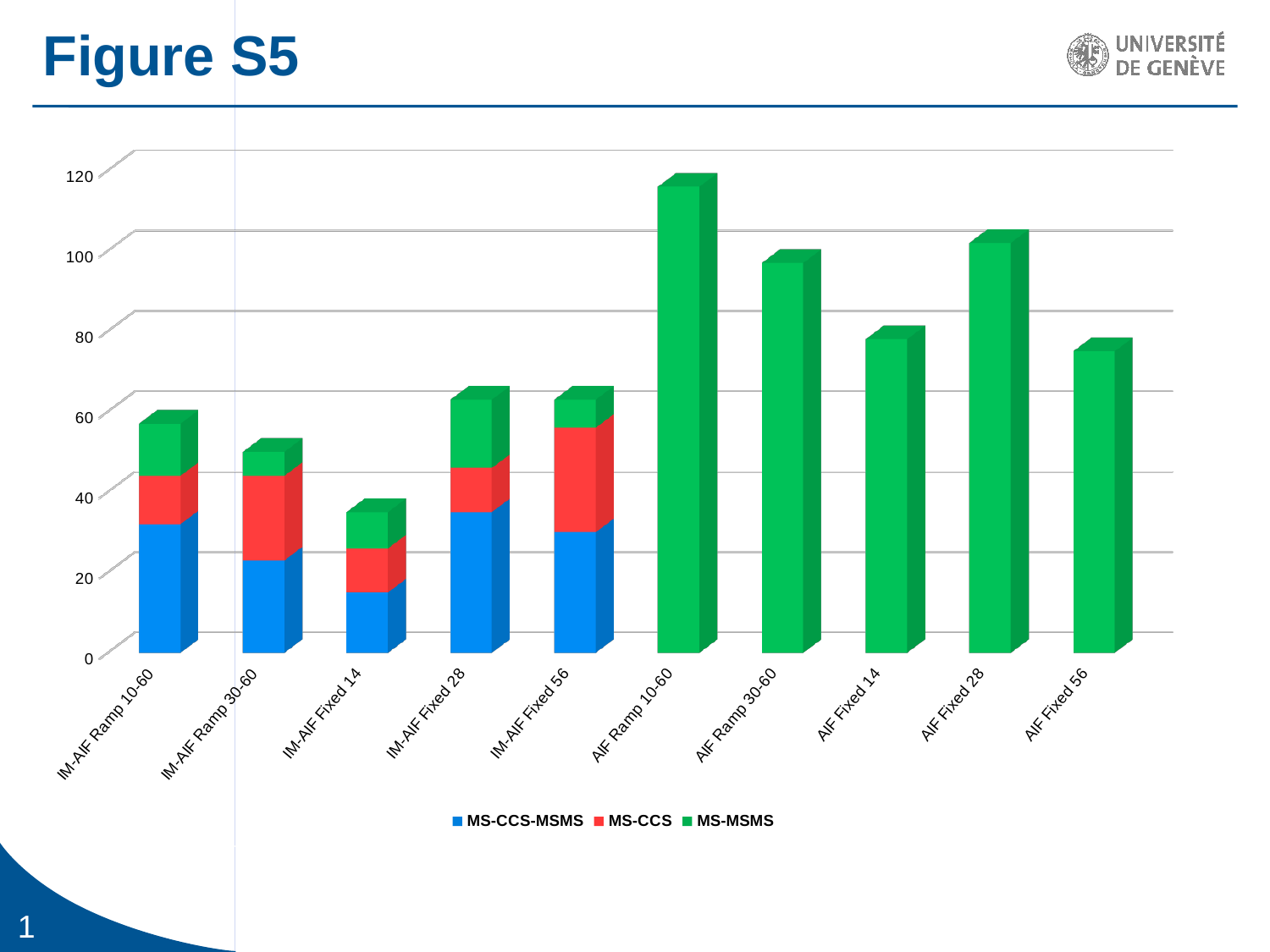

Figure S5
[unsupported chart]

Supplement: Supplementary file 1 [file metabolites-10-00464-s001.zip › Supplementary Material/Figure S5.pptx]

## Slide 1
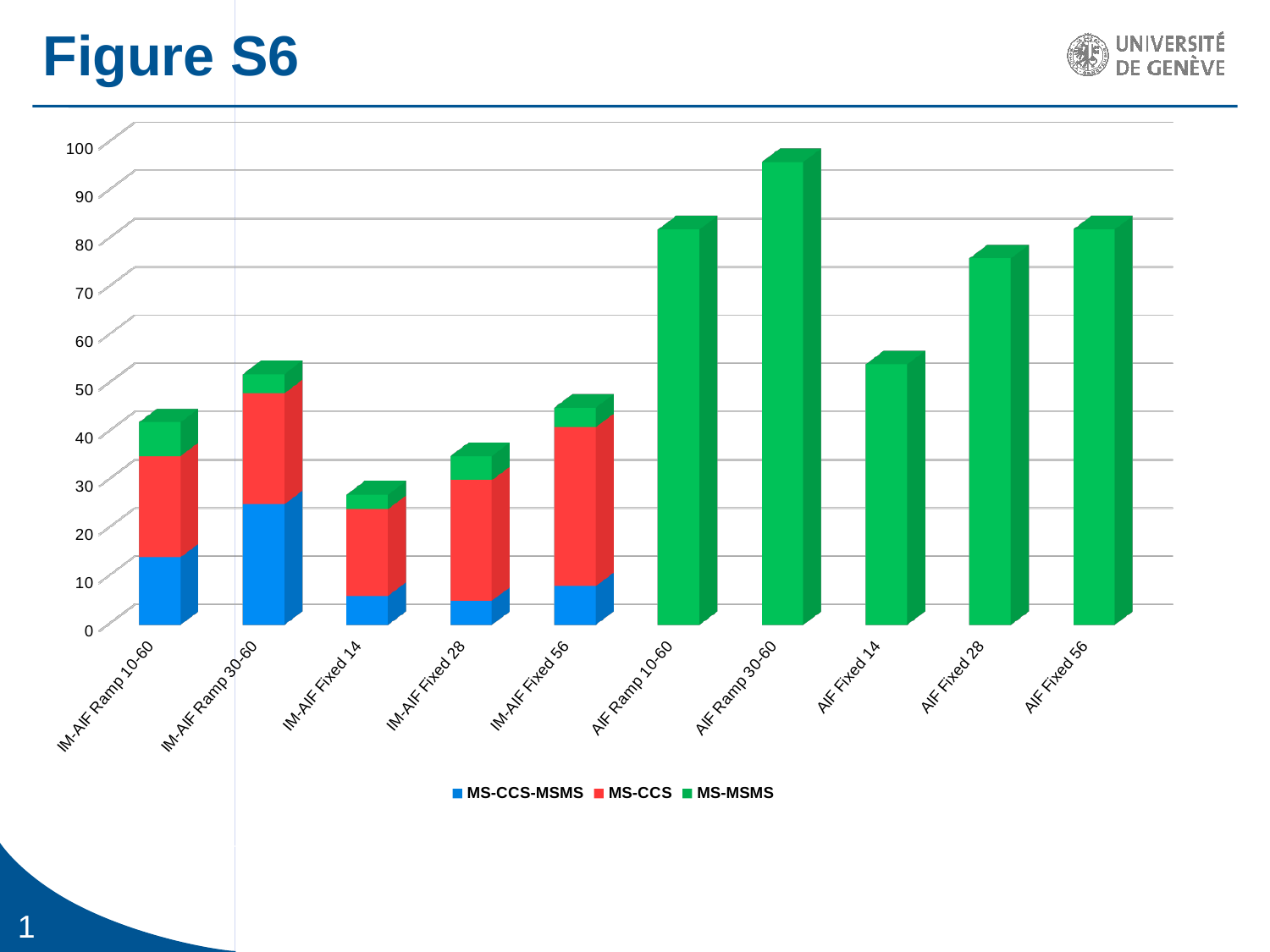

Figure S6
[unsupported chart]

Supplement: Supplementary file 1 [file metabolites-10-00464-s001.zip › Supplementary Material/Figure S6.pptx]

## Slide 1
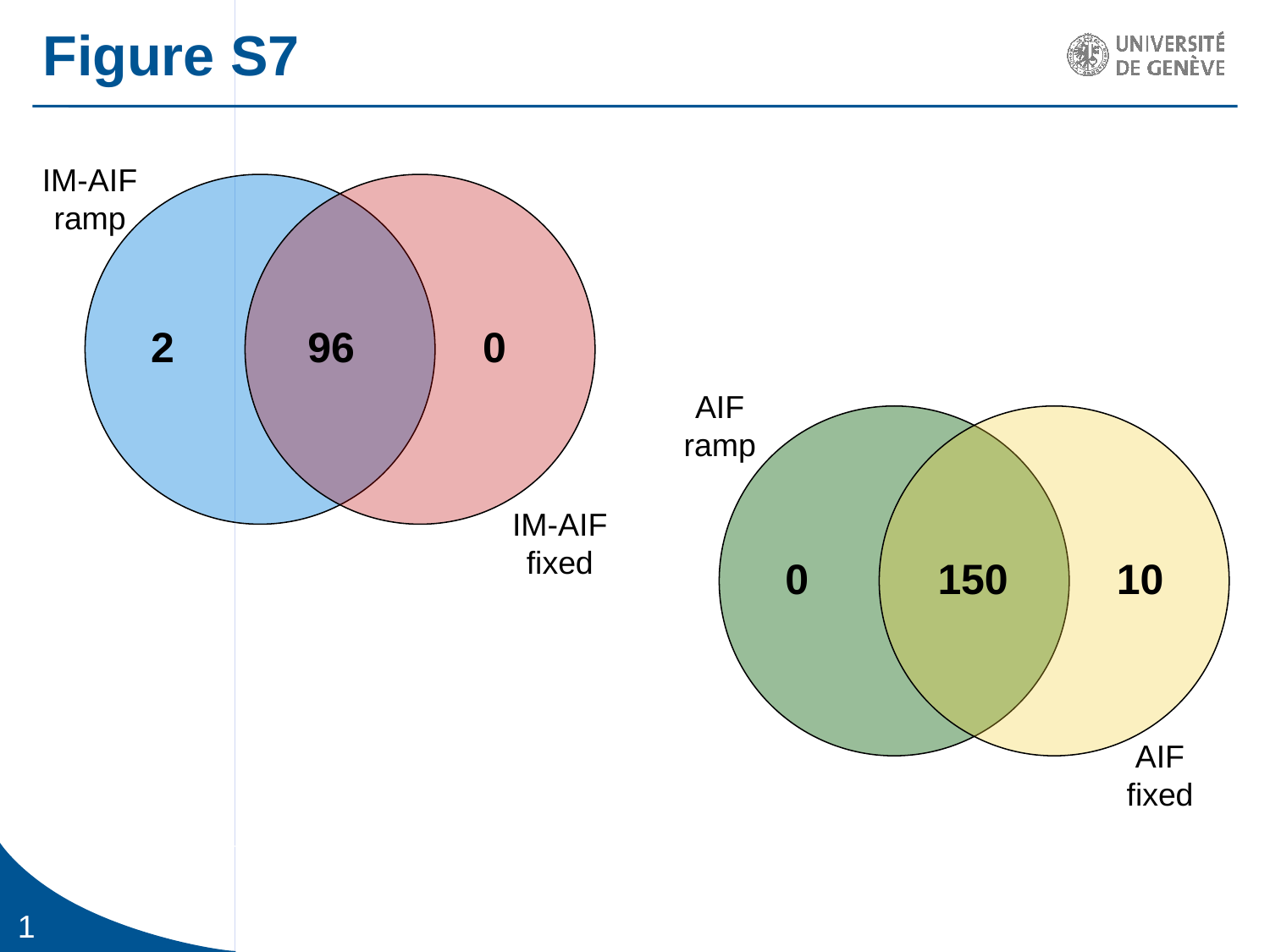

Figure S7
IM-AIF
ramp
2
0
96
AIF
ramp
IM-AIF
fixed
0
10
150
AIF
fixed

Supplement: Supplementary file 1 [file metabolites-10-00464-s001.zip › Supplementary Material/Figure S7.pptx]

## Slide 1
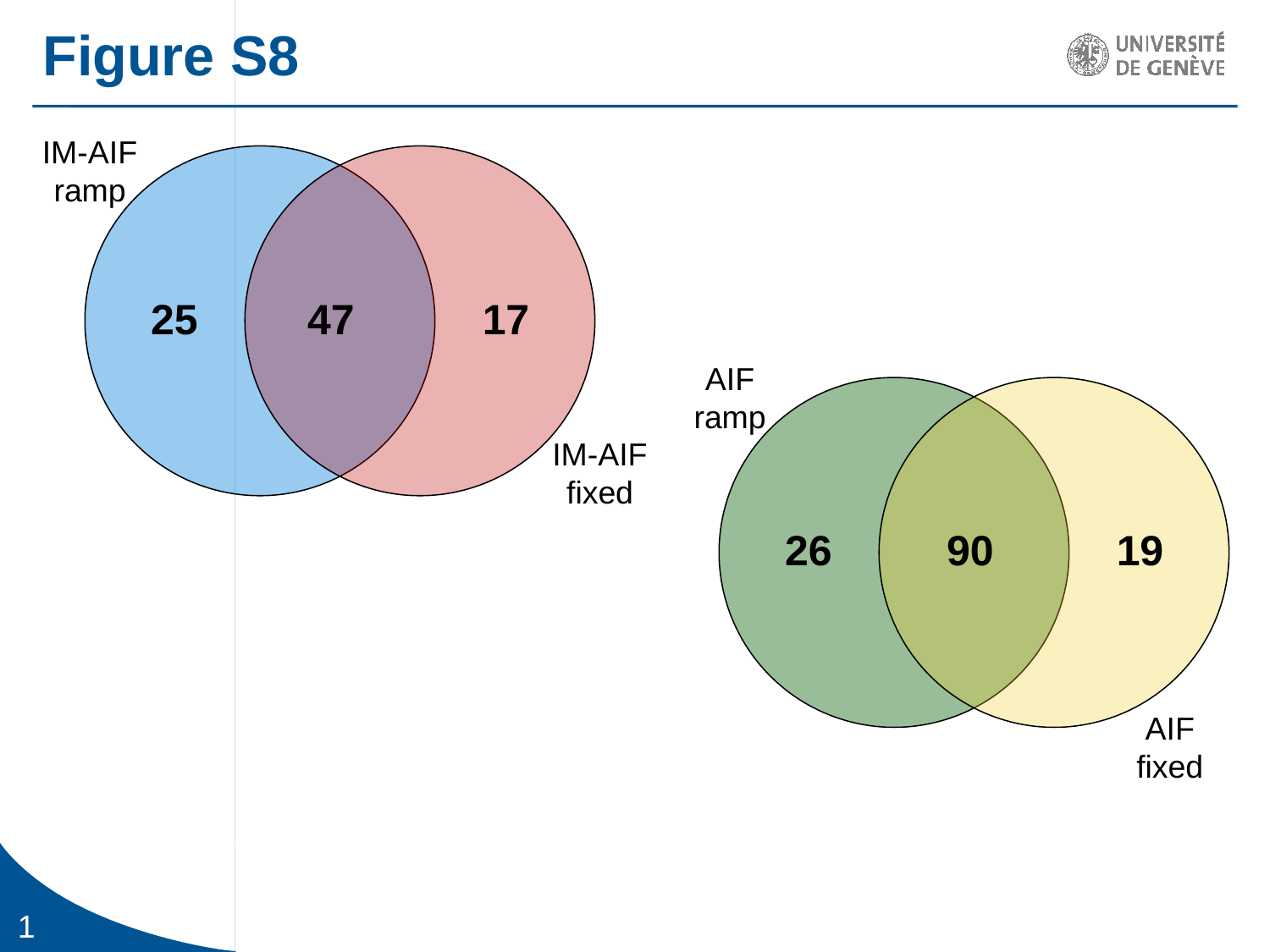

Figure S8
IM-AIF
ramp
25
17
47
AIF
ramp
IM-AIF
fixed
26
19
90
AIF
fixed

Supplement: Supplementary file 1 [file metabolites-10-00464-s001.zip › Supplementary Material/Figure S8.pptx]

## Slide 1
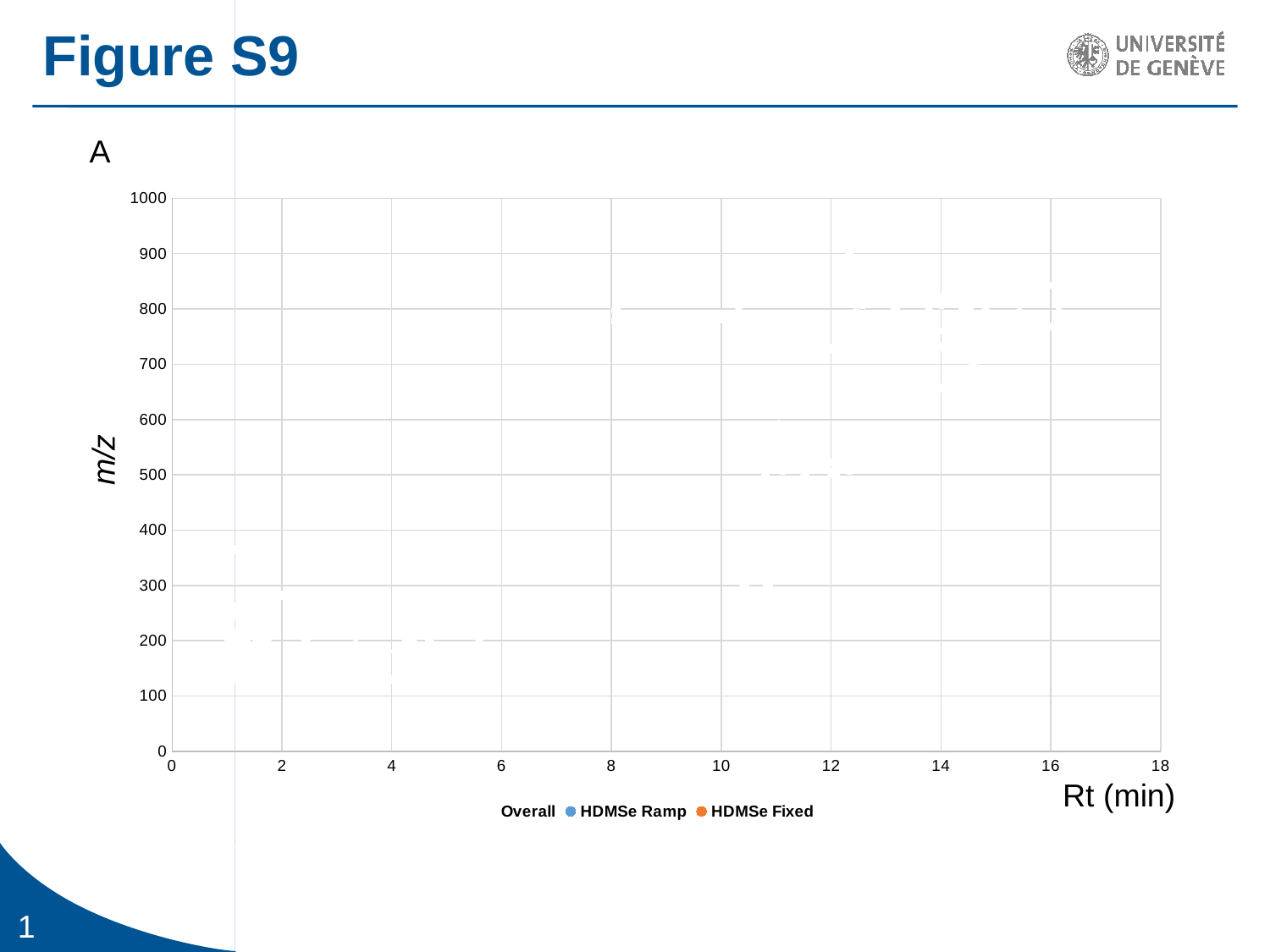

Figure S9
A
### Chart
| Category | | | |
|---|---|---|---|m/z
Rt (min)

## Slide 2
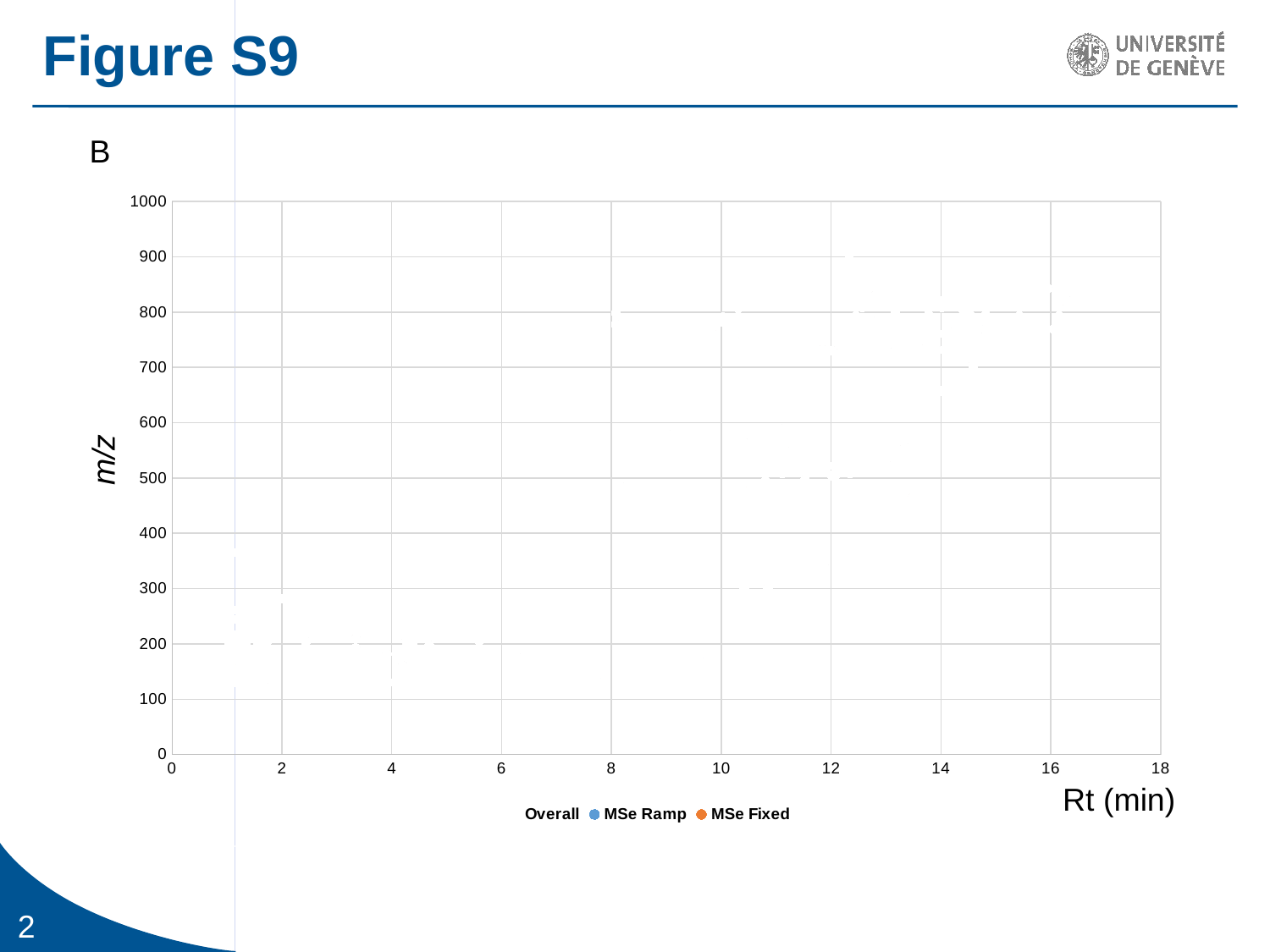

Figure S9
B
### Chart
| Category | | | |
|---|---|---|---|m/z
Rt (min)

Supplement: Supplementary file 1 [file metabolites-10-00464-s001.zip › Supplementary Material/Figure S9.pptx]
